# Snf1/AMP-activated protein kinase activates Arf3p to promote invasive yeast growth via a non-canonical GEF domain

**DOI:** 10.1038/ncomms8840

**Published:** 2015-07-22

**Authors:** Jia-Wei Hsu, Kuan-Jung Chen, Fang-Jen S. Lee

**Affiliations:** 1 Institute of Molecular Medicine, College of Medicine, National Taiwan University, Taipei 100, Taiwan; 2 Department of Medical Research, National Taiwan University Hospital, Taipei 100, Taiwan

## Abstract

Active GTP-bound Arf GTPases promote eukaryotic cell membrane trafficking and cytoskeletal remodelling. Arf activation is accelerated by guanine nucleotide-exchange factors (GEFs) using the critical catalytic glutamate in all known Sec7 domain sequences. Yeast Arf3p, a homologue of mammalian Arf6, is required for yeast invasive responses to glucose depletion. Here we identify Snf1p as a GEF that activates Arf3p when energy is limited. *SNF1* is the yeast homologue of AMP-activated protein kinase (AMPK), which is a key regulator of cellular energy homeostasis. As activation of Arf3p does not depend on the Snf1p kinase domain, assay of regulatory domain fragments yield evidence that the C-terminal hydrophobic α-helix core of Snf1p is a non-canonical GEF for Arf3p activation. Thus, our study reveals a novel mechanism for regulating cellular responses to energy deprivation, in particular invasive cell growth, through direct Arf activation by Snf1/AMPK.

## Abstract (web summary)

Snf1p is the yeast homologue of AMP-activated protein kinase, a key regulator of cellular energy homeostasis. Here, Hsu *et al.* identify Snf1p as a non-canonical guanine nucleotide-exchange factor for the Arf3p GTPase, regulating the yeast invasive response to glucose depletion.

## Graphical abstract

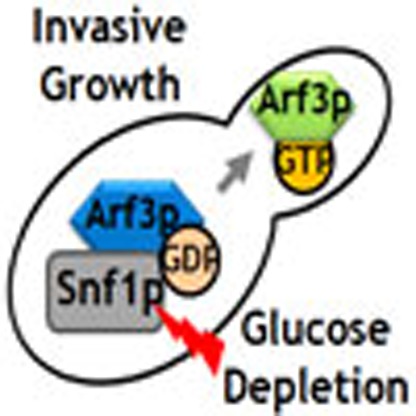

ADP-ribosylation factors (Arfs) are critical molecular switches involved in vesicle transport and actin reorganization through conformational changes controlled by their nucleotide-binding status^1^,^2^. Three Arf isoforms are expressed in the yeast *Saccharomyces cerevisiae*, whereas six were identified in mammalian cells. Based largely on sequence homology, the mammalian Arf family can be divided into three classes^3^: class I (Arf1-3), class II (Arf4-5) and class III (Arf6). Arf classes I and II primarily regulate vesicular trafficking between the Golgi and endoplasmic reticulum. Arf6 has been implicated specifically in endocytosis, plasma membrane protein recycling and cytoskeleton remodelling. Similar to other small GTP-binding proteins, the activation of Arfs is tightly regulated by guanine nucleotide-exchange factors (GEFs), which facilitate the dissociation of GDP and its replacement with GTP^4^. Although divergent in their overall sequences, all Arf GEFs are characterized by a ∼200 amino acids central catalytic domain referred to as the Sec7 domain, based on its homology to yeast Sec7p (refs 5, 6).

Living organisms utilize different strategies to cope with stress such as nutrient deprivation. Many fungi, such as *S. cerevisiae*, switch to filamentous growth to facilitate the foraging of nutrients when they become scarce^7^,^8^. The filamentous growth of some *S. cerevisiae* haploid strains is considered invasive, as colonies can penetrate into agar^9^. Yeast Arf3p is the homologue of mammalian Arf6, which is involved in multiple cellular processes, including cell adhesion, migration, wound healing, membrane ruffling and metastasis^1^. Recently, we reported that Arf3p is required for yeast invasive growth and that Arf3p activity is stimulated on glucose depletion^10^. Arf3p is also involved in polarity development in yeast^11^,^12^. During this process, Arf3p activation is mediated by Yel1p, which acts as the GEF^13^,^14^. However, this recently known Arf3 GEF is not responsible for the activation of Arf3p on glucose deprivation nor are other known yeast Arf GEFs^10^.

In this study, we report that Snf1p, the yeast homologue of AMP-activated protein kinase (AMPK), is a novel GEF required for Arf3p activation in response to glucose depletion. *SNF1*/AMPK is a key metabolic regulator of energy homeostasis and is involved in yeast invasive growth. We identified a non-canonical GEF domain in the conserved regulatory domain of Snf1p that directly binds to and activates yeast Arf3p. Surprisingly, Arf3p activation is independent of Snf1p kinase activity. Notably, we found that mutations in the regulatory domain result in markedly reduced Arf3p activation and invasive growth. These results reveal an unexpected function of Snf1/AMPK in yeast invasion through Arf GEF activity in response to glucose deprivation.

## Results

### Snf1p regulates Arf3p activation on glucose depletion

Arf3p is activated on glucose deprivation in yeast; however, none of the currently known Arf GEFs are responsible for this activation^10^. To identify the unknown Arf3p GEF, we used dominant-negative GDP-bound Arf3p^T31N^ to capture potential GEFs from lysates prepared from starved yeast. Four known GEFs for small GTPases (Lte1p, Rom2p, Gea1p and Sec7p) were identified from our mass spectroscopy analyses; however, we found that none of them were required for Arf3p activation during glucose depletion, as assessed by an effector pull-down assay that detects the activated form of Arf3p in cells (Supplementary Fig. 1a–d).

**Figure 1:**
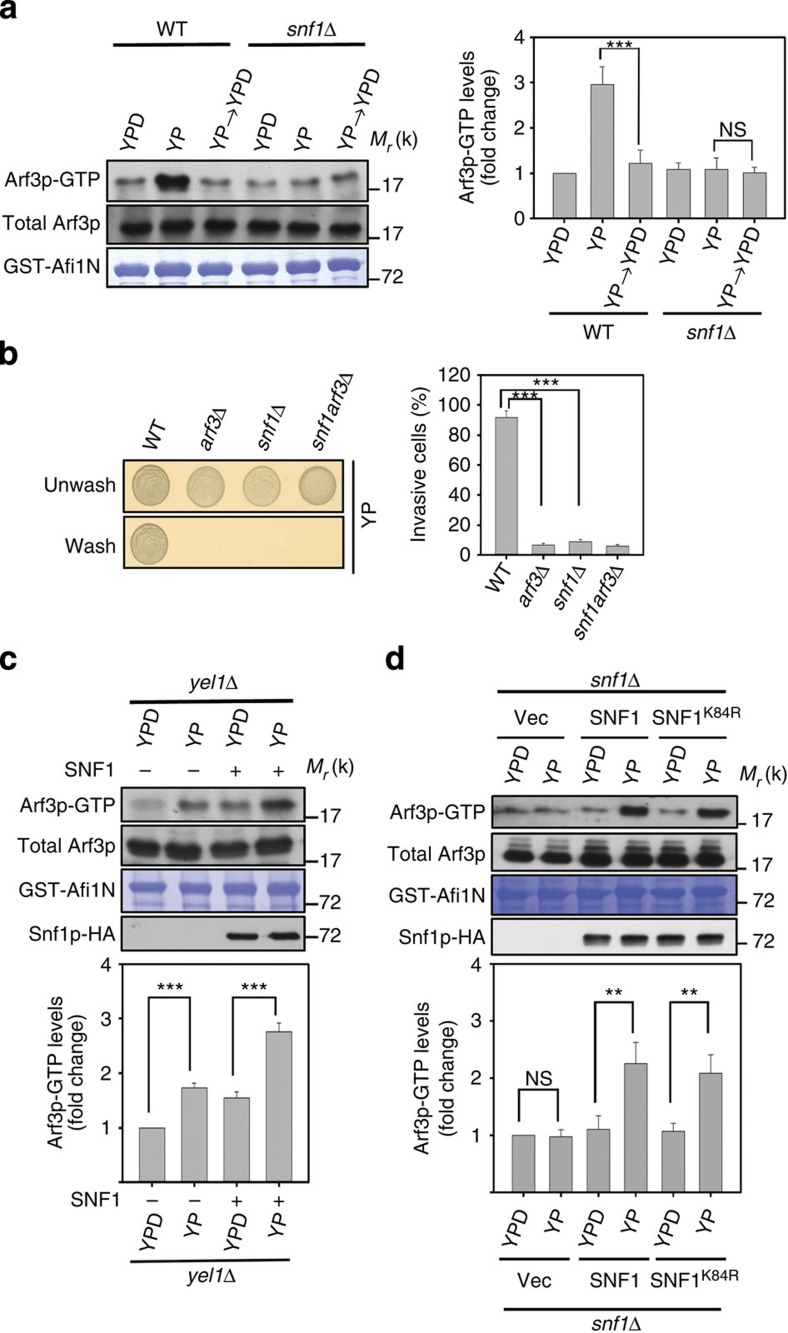
Snf1p regulates Arf3p activation independently of kinase activity in response to glucose depletion. (**a**) Yeast cells cultured in medium with glucose depletion (YP) or with glucose recovery (YPD) for 2 h were detected with GST-Afi1N and immunoblotted for Arf3p. Quantitative analysis of active Arf3p is presented in the right panel. Data are reported as the mean±s.d. of three experiments relative to wild type (WT) in YPD. ***P<0.001; Student's *t*-test. (**b**) Equal concentrations of the indicated Σ1278b yeast cells were spotted onto glucose-depleted (YP) plates and incubated for 16 h at 30 °C. Yeast invasion on YP plates was determined after rinsing with water. The percentage of invasive cells was quantified as described in Methods section. Data are reported as the mean±s.d. of three experiments. ***P<0.001; Student's *t*-test. (**c**,**d**) Active forms of Arf3p were precipitated by GST-Afi1N in *yel1*Δ cells expressing *SNF1* with YPD or YP treatment for 2 h (**c**) and in *snf1*Δ cells expressing *SNF1* and *SNF1*^K84R^ with YP treatment for 2 h (**d**). Quantitative analysis of active Arf3p is presented in the lower panels. Data are reported as the mean±s.d. of three experiments relative to (**c**) *yel1*Δ in YPD and (**d**) vector (Vec). **P<0.01 and ***P<0.001; Student's *t*-test.

Then, because our initial screening also identified a serine/threonine kinase Snf1p (for sucrose non-fermenting), the homologue of mammalian AMPK, as a binding partner of Arf3p^T31N^ in response to glucose depletion, we next investigated the role of this observed interaction. First, we found that Snf1p is critical for glucose depletion-induced Arf3p activation (Fig. 1a), and, in comparison, that the glucose sensing G-protein-coupled receptor Gpr1p is dispensable (Supplementary Fig. 1e). Arf3p activity is tightly regulated by glucose in the growth medium, and glucose-sensitive Arf3p activation depends on the presence of Snf1p (Fig. 1a). Consistent with these findings, both Arf3p and Snf1p were also required for glucose depletion-induced agar invasion (Fig. 1b); however, deletion of *SNF1,* but not *ARF3,* in yeast showed a pronounced growth defect on raffinose medium (Supplementary Fig. 2a). Strikingly, Snf1p overexpression enhanced Arf3p activation in both wild-type and *yel1*Δ cells (Supplementary Fig. 3a and Fig. 1c). This result indicates that Snf1p activates Arf3p independent of Yel1p, the only known Arf3p GEF to date.

Snf1p kinase activity is regulated by phosphorylation^15^. Three kinases, Tos3, Sak1 and Elm1, have overlapping and redundant functions for Snf1p activation, and deletion of all three kinases is required to abolish Snf1p activity *in vivo*^16^. We next tested whether Snf1p needs to be phosphorylated for the activation of Arf3p and found that Arf3p-GTP levels were much lower in *tos3sak1elm1*Δ cells than in wild type in response to glucose depletion (Supplementary Fig. 3b). We further examined whether the kinase activity of Snf1p is involved in Arf3p activation. Interestingly, we found that a kinase-dead Snf1p^K84R^ mutant^17^, which could not complement the growth defect of *snf1*Δ on raffinose medium (Supplementary Fig. 2b), could activate Arf3p in response to glucose depletion (Fig. 1d). These results suggest that Snf1p needs to be phosphorylated to activate Arf3p, but that activation is independent of Snf1p kinase activity.

### Snf1p interacts directly with Arf3p

**Figure 2:**
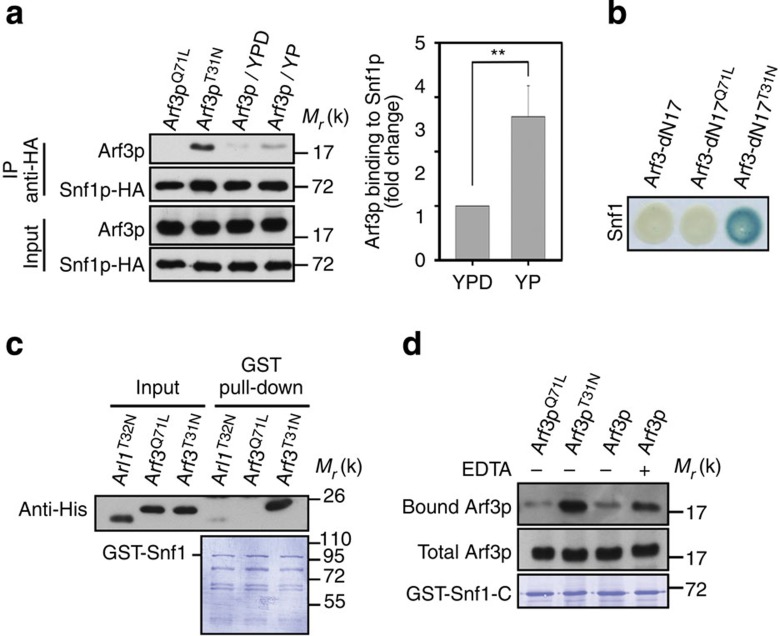
Snf1p interacts with Arf3p in response to glucose depletion. (**a**–**d**) Snf1p and Arf3p interaction. (**a**) Yeast cells were subjected to glucose depletion for 2 h; whole lysates were immunoprecipitated (IP) with anti-HA antibodies and immunoblotted for Arf3p. Data are reported as the mean±s.d. of three experiments relative to YPD. **P<0.01; Student's *t*-test. (**b**) Interactions between Snf1p and Arf3p were detected by yeast two-hybrid analysis as described in Methods section. (**c**) *In vitro* binding assay for recombinant Snf1 and Arf3 interaction. Purified GST-Snf1 from *E. coli* was incubated with recombinant His-Arf3^Q71L^, His-Arf3^T31N^ or His-Arl1^T32N^ for 1 h. The proteins were pulled down with glutathione-Sepharose 4B beads and visualized via immunoblotting with an anti-His antibody. (**d**) Yeast cells were lysed in the absence or presence of EDTA, and proteins from the cell lysates were precipitated by GST-Snf1-C and immunoblotted for Arf3p.

We next examined how Snf1p achieves Arf3p activation. First, we characterized the association between Arf3p and Snf1p, and found that dominant-negative Arf3p^T31N^, but not constitutively active Arf3p^Q71L^, co-immunoprecipitated with Snf1p (Supplementary Fig. 4a). Moreover, glucose depletion enhanced the interaction between Snf1p and Arf3p (Fig. 2a and Supplementary Fig. 4b). We then considered whether Snf1p could be divided into two domains, N-terminal kinase domain and C-terminal regulatory domain^18^ (Supplementary Fig. 5a). We found that the latter domain (Snf1-C) is sufficient to interact with Arf3 (Supplementary Fig. 5c–e). We also found that the kinase-dead Snf1p^K84R^ mutant could associate with Arf3p (Supplementary Fig. 5b). Thus, these results suggested that Snf1p utilizes its regulatory domain to associate with inactive Arf3p on glucose deprivation. We also performed a yeast two-hybrid analysis and *in vitro* binding assays, which revealed that recombinant Snf1p interacts directly and specifically with inactive Arf3p, but not with another Arf-like protein, Arl1 (Fig. 2b,c). To examine whether nucleotide binding is necessary for the observed interaction, we used EDTA to chelate magnesium, a divalent cation required for nucleotide binding^19^, to generate largely nucleotide-free Arf3p (ref. 20). We found that more Arf3p was associated with Snf1-C in the presence of EDTA (Fig. 2d). Thus, the direct interaction and preference for binding nucleotide-free Arf3p led us to hypothesize that Snf1p might act as a GEF towards Arf3p.

### Snf1p acts as an Arf3p GEF on glucose depletion

**Figure 3:**
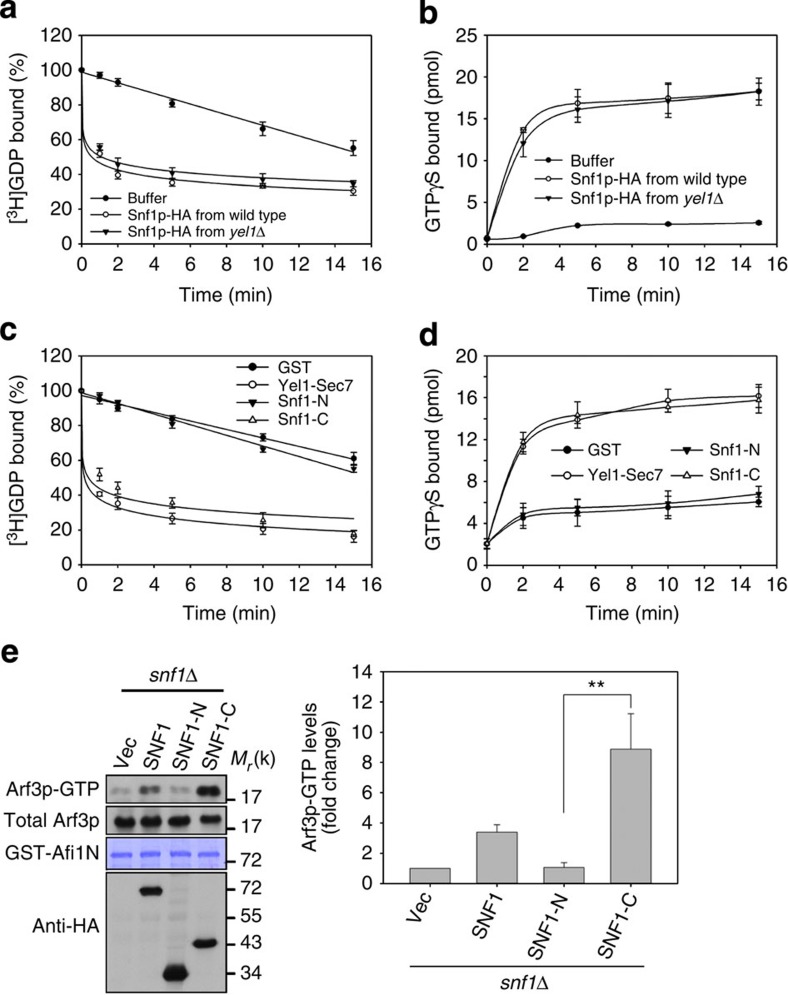
C-terminal regulatory domain of Snf1p activates Arf3p. (**a**–**d**) Activation of Arf3p by Snf1p. [^3^H]GDP dissociation from and [^35^S]GTPγS binding to Arf3-dN17 in the presence of Snf1p immunoprecipitated from wild-type and *yel1*Δ cells (**a**,**b**), or recombinant Yel1-Sec7, Snf1-N and Snf1-C (**c**,**d**), were monitored by measuring radioactivity. The data are reported as the means±s.d. of the percentages of dissociated [^3^H]GDP and of bound [^35^S]GTPγS (*n*=3). (**e**) Active forms of Arf3p were precipitated by GST-Afi1N in *snf1*Δ cells expressing *SNF1*, *SNF1-N* or *SNF1-C*. Right panel, quantitative analysis of active Arf3p. Data are reported as the mean±s.d. of three experiments relative to vector (Vec) control. **P<0.01; Student's *t*-test.

To test whether Snf1p possesses GEF activity, we first examined the guanine nucleotide-exchange activity of immuno-isolated Snf1p-HA from wild-type versus *yel1*Δ cells, and found that they both increased GDP dissociation from and GTPγS binding to recombinant Arf3p (Fig. 3a,b). We also purified recombinant Snf1 fragments to examine their GEF activity towards recombinant Arf3p *in vitro*, and found that Snf1-C, but not Snf1-N, catalysed the GDP release from and GTPγS loading of Arf3, which was comparable to the effect of the Sec7 domain of Yel1p (the currently known Arf3p GEF; Fig. 3c,d). Furthermore, Snf1-C did not interact with or accelerate the guanine nucleotide exchange of other Arf or Arf-like proteins, including Arf1, Arl1 and Arl3 (Supplementary Fig. 6a–c). Thus, the regulatory domain of Snf1 is a specific Arf3 GEF *in vitro*. Next, to examine whether Snf1p-C is sufficient to activate Arf3p *in vivo*, we overexpressed *SNF1-C* in *snf1*Δ cells and found that *SNF1-C,* but not *SNF1-N*, could activate Arf3p significantly (Fig. 3e). However, *SNF1-C* could not rescue the *snf1*Δ growth defect on nutrient-limited medium (Supplementary Fig. 2c). This result is expected considering that Snf1p/AMPK should have many downstream targets, in addition to Arf3p, in mediating its role as a central coordinator of cellular energy homeostasis.

**Figure 4:**
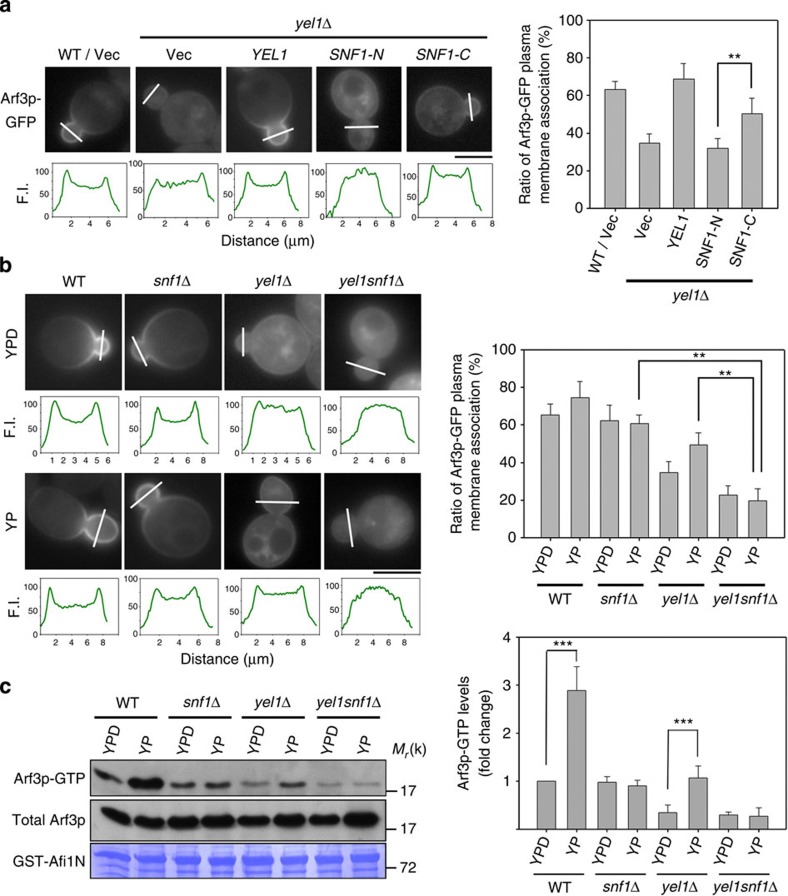
C-terminal regulatory domain of Snf1p is responsible for activating Arf3p in response to glucose depletion. (**a**) *ARF3-GFP* expressed under the control of the *ADH1* promoter (*CEN* plasmid) was transformed into the indicated yeast cells. Transformants were grown to the exponential phase and inspected via microscopy. Scale bar, 5 μm. (**b**) The localization of Arf3p-GFP was observed in the indicated yeast cells grown in YPD or YP medium for 2 h. Scale bar, 5 μm. The fluorescence intensity (F.I.) profiles from the line scan are shown in each lower panel. The plasma membrane association ratio of Arf3p was quantified using Axio Vision Rel. 4.2 software. The F.I.'s of the plasma membrane signals were summed and divided by the whole-cell signals (*n*=50) as described in Methods section. Data are reported as the mean±s.d. **P<0.01; Student's *t*-test. (**c**) Active forms of Arf3p were precipitated by GST-Afi1N in the indicated cells and immunoblotted for Arf3p. Quantitative analysis of active Arf3p is presented in the right panel. Data are reported as the mean±s.d. of three experiments. ***P<0.001; Student's *t*-test. Vec, vector; WT, wild type.

We next assessed the relative roles of the two Arf3p GEFs, Snf1p and Yel1p. The activation of Arf3p by Snf1p does not require the presence of Yel1p (Fig. 1c). Moreover, *SNF1-C* overexpression in *yel1*Δ cells increased the targeting of Arf3p to the plasma membrane, which is a signature effect of Arf3p activation in yeast^12^,^13^ (Fig. 4a). We also assessed the activation status of Arf3p in the different deletion strains by examining the distribution of Arf3p on the plasma membrane and the level of active Arf3p using effector pull-down assays (Fig. 4b,c). We found that *snf1*Δ cells could not increase Arf3p activation on glucose deprivation, whereas *yel1*Δ cells had this ability. Moreover, the level of active Arf3p was reduced in *yel1snf1*Δ cells compared with that in the single-deletion mutants. Thus, the results suggest that the two Arf3GEFs function independently in controlling Arf3p activation; both Snf1p and Yel1p activate Arf3p during glucose-starvation conditions, whereas only Yel1p activates Arf3p during vegetative growth.

### The regulatory sequence of Snf1p directly activates Arf3p

**Figure 5:**
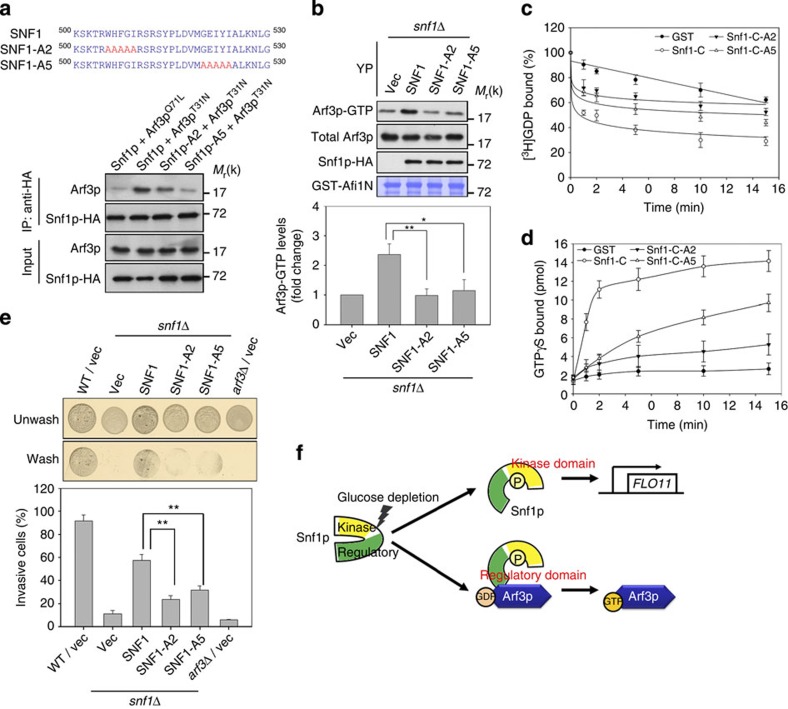
C-terminal RS of Snf1p interacts with and activates Arf3p. (**a**) Snf1p, Snf1p-A2, and Snf1p-A5 were immunoprecipitated (IP) with anti-HA antibodies, and the bound proteins were immunoblotted for the presence of Arf3p. (**b**) Arf3p-GTP forms were precipitated by GST-Afi1N in *snf1*Δ cells expressing Snf1p, Snf1p-A2 or Snf1p-A5. Below, quantitative analysis of active Arf3p. Data are reported as the mean±s.d. of three experiments relative to a vector (Vec) control. *P<0.05 and **P<0.01; Student's *t*-test. (**c**,**d**) [^3^H]GDP dissociation (**c**) from and [^35^S]GTPγS binding (**d**) to Arf3 in the presence of Snf1-C, Snf1-C-A2 or Snf1-C-A5 were monitored by measuring radioactivity. Data are reported as the means±s.d. of the percentages of dissociated [^3^H]GDP and of bound [^35^S]GTPγS (*n*=3). (**e**) Σ1278b yeast cells containing a *SNF1* deletion transformed with different forms of *SNF1* (full length, A2 or A5) were spotted onto YP plates for 16 h to examine agar penetration. The percentage of invasive cells was quantified as described in Methods section. Data are reported as the mean±s.d. of three experiments. **P<0.01; Student's *t*-test. (**f**) A working model for Arf3p activation by Snf1p to mediate invasive growth in response to glucose depletion. Snf1p/AMPK utilizes its N-terminal kinase domain to regulate *FLO11* gene transcription and its atypical Arf GEF at the C-terminal regulatory domain to promote Arf3p activation in response to glucose deprivation.

To gain further insight into how Snf1 acts as an Arf GEF, we next dissected the regulatory domain of Snf1p into Snf1-C1 (amino acid (a.a.) 392–518) and Snf1-C2 (a.a. 515–633), which are regions that interact with the γ- and β-subunits of the kinase, respectively^21^,^22^. We found that Snf1-C1 is sufficient to interact with Arf3^T31N^ (Supplementary Fig. 7a). Snf1-C1 contains auto-inhibitory sequence (AIS) and regulatory sequence (RS) that have been reported to repress Snf1p kinase activity^22^. Although Snf1-C1 did not share sequence similarity with canonical Sec7 domains, sequence alignment of the C1 region of Snf1p/AMPK homologues from different eukaryotes revealed that this region is evolutionarily conserved (Supplementary Fig. 7b). To identify the interaction interface, we utilized alanine scanning to examine interactions within regions of the AIS and RS (Supplementary Fig. 7c). None of the alanine mutants in the AIS disrupted the interaction between Snf1p and Arf3p. In contrast, the RS mutant Snf1p-A5 (a.a. 520–524) showed defective binding to Arf3p^T31N^ (Supplementary Fig. 8a and Fig. 5a). Consistent with its failure to bind to Arf3p, expression of *SNF1-A5* in *snf1*Δ cells could neither activate Arf3p (Supplementary Fig. 8b and Fig. 5b) nor restore yeast invasion (Fig. 5e). Another mutant, *SNF1-A2* (a.a. 505–509), also showed defective Arf3 GEF activity as measured by the GDP dissociation (Fig. 5c) and GTPγS binding (Fig. 5d) of Arf3, even though it could still interact with Arf3p (Fig. 5a). Similarly, *SNF1-A2* could not support Arf3p activation (Supplementary Fig. 8b and Fig. 5b) and yeast invasive growth (Fig. 5e) in *snf1*Δ cells. However, expression of Snf1p-A2, unlike Snf1p-A5, did not rescue the raffinose hypersensitivity of *snf1*Δ cells (Supplementary Fig. 8c), even though both mutants could be phosphorylated in response to glucose depletion (Supplementary Fig. 8d). These results indicate that Snf1p-A2 may contain both the catalytic residues for Arf3p activation and the critical amino acids for its transcriptional regulatory activity. Taken together, these findings suggest that the C-terminal α-helix hydrophobic core of Snf1p is responsible for the Arf3p GEF activity that becomes activated during glucose depletion-induced yeast invasion.

### Arf3p is dispensable for Snf1p-dependent *FLO11* expression

**Figure 6:**
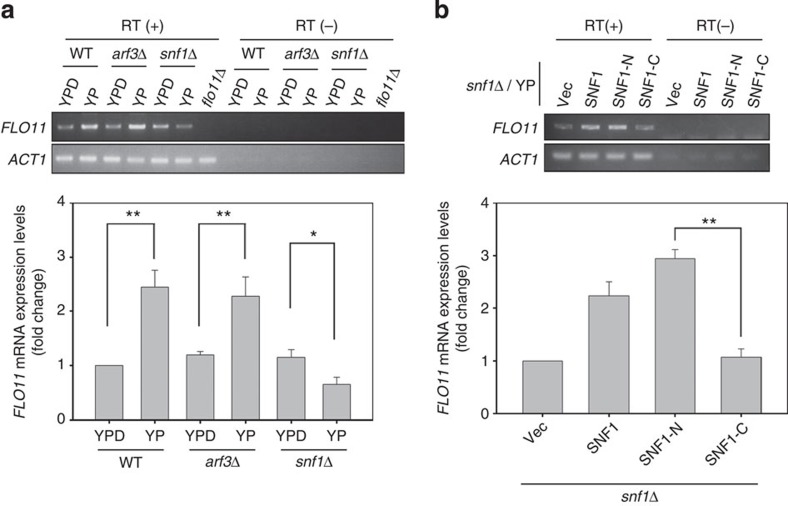
Regulation of *FLO11* mRNA transcription by Snf1. (**a**,**b**) *FLO11* mRNA detection in indicated yeast cells (**a**) in response to glucose depletion or (**b**) in *snf1*Δ cells with the expression of *SNF1*, *SNF1-N* or *SNF1-C*. Total RNA of indicated cells grown in YPD or YP were isolated and used as the template for reverse transcription (RT) PCR (RT (+)). PCRs only, as negative control, are indicated as RT (−). The level of RT–PCR products of *FLO11* mRNA was quantitated and data are reported as the mean fold change±s.d. of three experiments relative to (**a**) wild type (WT) in YPD and (**b**) vector (Vec; lower panels). *P<0.05 and **P<0.01; Student's *t*-test.

*SNF1* is best characterized by its transcriptional activation mediated via its kinase activity^18^. Notably, one of its downstream functions is the induction of surface glycoprotein *FLO11* mRNA production to promote invasive growth^23^. Unlike in *snf1*Δ cells, we found that the *FLO11* mRNA level increased in *arf3*Δ cells in response to glucose deprivation (Fig. 6a). Moreover, the expression of the Snf1p kinase domain (*SNF1-N*), but not the regulatory domain (*SNF1-C*), in *snf1*Δ cells significantly increased *FLO11* mRNA levels (Fig. 6b). Thus, Snf1p controls multiple effector pathways in response to nutrient depletion: Snf1 uses its N-terminal kinase domain to regulate certain effector events, such as *FLO11* gene transcription, and uses its C-terminal regulatory domain to couple to other events, such as Arf3p activation.

## Discussion

Snf1/AMPK has multiple regulatory targets to mediate different responses when energy is limited^18^, including the transcriptional activation of the *FLO11* gene for yeast invasive growth^23^. In this study, we present a novel role of Snf1p in modulating nutrient limitation. Independent of Snf1p N-terminal kinase activity, evidence regarding the physical and genetic relationship of Snf1p to Arf3p suggests that Snf1p also promotes yeast invasive growth by activating Arf3p through its C-terminal GEF activity.

Thus far, all identified Arf GEF molecules are characterized by a central catalytic Sec7 domain^2^,^6^, and the Sec7 domain alone is sufficient for the catalysis of nucleotide exchange on Arfs. Our findings now challenge this consensus view. We identified a non-canonical GEF domain in Snf1/AMPK that is both necessary and sufficient for Arf activation and that does not resemble the Sec7 domain. Crystal structures of the Snf1p regulatory domain^24^ and conventional Sec7 domain^25^ have been determined. The structure of a Sec7 domain GEF is an exclusively elongated α-helical protein^25^. However, the central component of the Snf1p regulatory domain is primarily anti-parallel β-sheet^24^. Our findings demonstrated that the only α-helix hydrophobic core of the Snf1p regulatory domain (a.a. 520–524) is responsible for the regulation of Arf3p interaction and activity in response to glucose depletion-induced yeast invasion. These results may provide new insight into different mechanisms of activating Arf GTPases. Moreover, the Rab1 GTPase was shown to be activated by >1 mechanistically distinct GDP-release pathway that depended on the nature of its cognate GEF^26^. Further structural and biochemical analysis are needed to identify the mechanisms of Arf activation used by different Arf GEF domains.

The Snf1/AMPK is activated by various cellular stresses such as glucose limitation. The kinase activity increases when a conserved threonine residue in the activation loop is phosphorylated by upstream kinases^17^. We found that activation of Snf1p by its upstream kinases is required for the activation of Arf3p and excluded the possibility that Snf1p kinase activity activates Arf GTPases; however, how the signal of glucose starvation is transduced to activate the regulatory domain of Snf1/AMPK as an Arf GEF requires further investigation. In addition, Snf1/AMPK exists as an obligate heterotrimer, containing a catalytic subunit (α) and two regulatory subunits (β and γ; refs 18, 27). The yeast genome encodes the catalytic α-subunit Snf1p, three alternative β-subunits (Sip1p, Sip2p and Gal83p) and the γ-subunit Snf4p. The function of the γ-regulatory subunit is to control Snf1/AMPK kinase activity in both yeast and mammals. In glucose-grown cells, the C-terminal regulatory domain of Snf1p auto-inhibits the catalytic kinase domain, whereas the Snf4 subunit binds to the Snf1p regulatory domain and counteracts this auto-inhibition in glucose-deprived cells. In addition, the C-terminal region of Snf1/AMPK is required for the formation of a complex of the β- and γ-subunits^22^. Furthermore, the mammalian DOCK180 and ELMO1 complex was shown to function as a bipartite GEF in which DOCK180 interacts with Rac1 and ELMO1 potentiates DOCK180-dependent Rac activation^28^. *In vitro*, the C-terminal atypical Arf GEF domain of Snf1p purified from bacteria is sufficient for Arf3 binding and GTP loading. Although these results are important to demonstrate the exchange activity of the C-terminal atypical Arf GEF domain, they may need to be reconciled with the dependence of other subunits for Arf3p activation *in vivo*. Thus, the control of α-subunit-mediated Arf GTPases activation by the regulatory subunits of the Snf1/AMPK heterotrimer remains a critical area for future research.

In summary, our results reveal a surprising role for Snf1/AMPK as a novel Arf GEF. We find that this role enables yeast invasive growth that is induced as an adaptive response to nutrient deprivation. Our study reveals that Snf1/AMPK utilizes its N-terminal kinase domain to regulate gene transcription and its C-terminal atypical Arf GEF domain to mediate polarized invasive growth under conditions of nutrient limitation (Fig. 5f). Our findings also provide a hitherto missing component in our understanding of Arf-dependent invasive cell growth.

## Methods

### Plasmids and yeast culture

Standard protocols were used to generate Σ1278b yeast strains via homologous recombination^29^. All of the yeast *ARF3* plasmids used here encode Arf3p constructs driven by the native *ARF3* promoter. All yeast strains and plasmids used in this study are outlined in Supplementary Tables 1 and 2. For glucose depletion, Σ1278b yeast cells were cultured in YPD (yeast extract/peptone/dextrose) overnight, washed three times with ddH_2_O, re-cultured in YP medium or spotted on YP agar plates without adding any other carbon source, and incubated for 2 or 16 h.

### *In vitro* binding assay

*Escherichia coli* strain BL21 (DE3; Novagen, La Jolla, CA) was transformed with plasmids including pET32a-ARF3 (Q71L or T31N), pGEX4T-1 and pGEX4T-1 containing various *SNF1* fragments, as shown in Supplementary Fig. 2a. After the cells were induced with 0.5 mM isopropyl β-D-1-thiogalactopyranoside at 37 °C for 3 h, glutathione *S*-transferase (GST) fusion proteins or His-tagged proteins were purified from *E. coli* lysates using glutathione-Sepharose 4B (GE Healthcare Amersham, Piscataway, NJ) or nickel affinity resin (Qiagen, Valencia, CA), respectively. In the pull-down assays, GST, GST-Snf1-C (a.a. 311–633), GST-Snf1-C1 (a.a. 392–518) and GST-Snf1-C2 (a.a. 515–633) were immobilized on glutathione beads and incubated with His-Arf3 (Q71L or T31N) with GDP or GTPγS loaded in binding buffer (50 mM Tris-HCl (pH 7.5), 150 mM NaCl, 1% Triton X-100, 1 mM dithiothreitol (DTT) and 10 mM MgCl_2_) for 1 h at 4 °C. Then, the beads were washed three times with 1 ml of binding buffer. Next, bound proteins were analysed by western blotting using anti-His monoclonal antibodies (BD Biosciences, La Jolla, CA, no. 552565; 1:5,000).

### Yeast two-hybrid assay

The yeast strain YEM1α was co-transformed with different combinations of bait (pEG202) and prey (pJG4-5) plasmids^30^. The reporter yeast YEM1α, expressing interacting proteins, can transactivate two reporter genes, *LacZ* and *LEU2*, allowing for the expression of β-galactosidase and for growth on minimal medium lacking leucine.

### Immunoprecipitation

For the yeast Snf1p and Arf3p interaction, Σ1278b yeast cells co-expressing Arf3p and Snf1p-HA were disrupted with glass beads in extraction buffer (PBS containing 1 mM DTT, 5 mM MgCl_2_ and protease inhibitors). The extracts were cleared via centrifugation at 4,000 r.p.m. for 10 min. Agarose beads conjugated with a monoclonal anti-hemagglutinin (anti-HA) antibody (mouse monoclonal anti-HA agarose antibody; Sigma, St Louis, MO, no. A2095; 1:200) were added to the cleared extracts and incubated at 4 °C for 2 h. Then, the beads were washed three times with wash buffer (extraction buffer containing 0.5% Triton X-100), and the bound complexes were eluted with sample buffer. The bound proteins were subjected to SDS–polyacrylamide gel electrophoresis and analysed via western blotting.

### Viability assay

For the raffinose treatments, 10-fold serial dilutions of mid-log phase yeast cells were spotted onto YPD plates or YP plates with 2% raffinose. The plates were grown for 3 days before imaging.

### *FLO11* mRNA detection by reverse transcription–PCR

For steady-state mRNA expression analysis, total RNA was prepared by the hot acid phenol method^31^. cDNA was prepared from equal amounts of total RNA from each sample using a RevertAid H Minus First Strand cDNA Synthesis kit (Fermentas) following the manufacturer's instructions, and *FLO11* and *ACT1* mRNAs were detected by reverse transcription–PCR. Half of each reaction was separated on a 1.5% agarose gel and stained with ethidium bromide. The *FLO11* mRNA products were quantified and normalized to *ACT1* products using ImageJ software. The values represent the mean of three independent experiments.

### Microscopy

All images of living cells containing GFP-tagged proteins were obtained after growth in synthetic medium to mid-log phase. Fluorescence microscopy was performed using a Zeiss Axioskop microscope equipped with a Cool Snap FX camera. Cells were viewed at × 100 magnification. For all microscopic examinations, the exposure times and image processing procedures were identical for each sample within an experiment. The light levels were scaled equivalently among all samples within an experiment when the images were exported from the imaging software and when they were subsequently processed in Photoshop. Only the light level min/max settings were adjusted for clarity. The distance of moving fluorescence line scans and the arbitrary profile of intensity values were established using the Axio Vision Rel 4.2 software^13^. Quantification of plasma membrane association of Arf3p was calculated by measuring fluorescence signals of the plasma membrane (*P*) and the whole cell (*C*). The percentage of the total fluorescence of both areas was determined as *P*/*C* × 100%. Measurements were performed on images acquired with the same microscope intensity settings^11^.

### Invasive growth assay

The haploid cell invasive assay was performed by plate-washing method^10^. Briefly, equal concentrations of Σ1278b yeast cells were spotted onto YP plates. After incubation for 16 h at 30 °C, cells that penetrated the agar were examined after washing them off the agar surface with water. The samples were photographed before and after washing with a stream of water. To perform a quantitative agar invasion assay^10^, agar blocks containing yeast colonies (before and after washing) were excised. Elution buffer containing 0.5 M sodium acetate (pH 7.0) and 1 mM EDTA (pH 8.0) was added, and the tubes were incubated at 55 °C for 20 min until the agar blocks were completely dissolved. The yeast cell numbers were examined by measuring OD_600_ values to obtain total cell numbers before and after washing with water. The percentage of invasive cells observed after washing with water was determined as OD_600_ unit after washing/OD_600_ unit before washing × 100%. The data are reported as the mean±s.d. of three experiments.

### *In vitro* GEF-activity assays

Guanine nucleotide-exchange assays were performed by measuring the binding of GTPγS^32^,^33^. Briefly, 1 μM ARF proteins and 8 μM [^35^S]GTPγS in exchange buffer containing 50 mM HEPES (pH 7.5), 1 mM MgCl_2_, 100 mM KCl and 1 mM DTT were incubated with 100 nM GST, GST-Yel1-Sec7 or GST-fusion proteins containing various *SNF1* fragments. Samples were collected at various time points, diluted with 1.2 ml of ice-cold stopping buffer (50 mM HEPES (pH 7.5), 10 mM MgCl_2_, 100 mM KCl and 1 mM DTT) and filtered on membrane filter (MF) membrane nitrocellulose filters (Millipore). The filters were washed, dried and counted in a liquid scintillation counter (Beckman, LS6000IC). For the GDP dissociation assays^34^,^35^, His-tagged ARF proteins (1 μM) were first loaded with 20 μM [^3^H]GDP for 50 min at 30 °C in exchange buffer. Spontaneous nucleotide dissociation and exchange were catalysed by the addition of 1 mM unlabelled GTP and 100 nM GST, GST-Yel1-Sec7 or GST-fusion proteins containing various *SNF1* fragments. After various incubation times, proteins and bound nucleotides were isolated by filtration through nitrocellulose filters and quantified by liquid scintillation counting.

### Active small GTPase pull-down assay

For the yeast Arf3p activity assay, Σ1278b yeast cells expressing *ARF3* were grown in rich medium containing 2% (YPD) or 0% (YP) glucose and were lysed with glass beads at 4 °C in lysis buffer (PBS containing 1 mM DTT, 5 mM MgCl_2_ and protease inhibitors (1 μg ml^−1^ aprotinin, 1 μg ml^−1^ leupeptin, 1 μg ml^−1^ pepstatin, 1 μM benzamidine and 1 mM phenylmethylsulfonyl fluoride (PMSF))). The lysates were centrifuged at 3,000 r.p.m. for 5 min, and the clarified lysates were incubated with 10 μg of GST-Afi1N bound to glutathione-Sepharose beads (GE Healthcare). The beads were washed three times with lysis buffer containing 0.2% Triton X-100, and the bound proteins were eluted with sample buffer. The samples were assayed for the presence of Arf3p by western blotting (1:3,000). Scans of uncropped blots are shown in Supplementary Figs 9–14.

### Statistical analysis

Statistical analysis was carried out using SigmaPlot v10.0 to assess differences between experimental groups. Statistical significance was analysed by Student's *t*-test and expressed as a *P* value.

## Additional information

**How to cite this article:** Hsu, J.-W. *et al.* Snf1/AMP-activated protein kinase activates Arf3p to promote invasive yeast growth via a non-canonical GEF domain. *Nat. Commun.* 6:7840 doi: 10.1038/ncomms8840 (2015).

## Supplementary Material

Supplementary Information Supplementary Figures 1-14, Supplementary Tables 1-2 and Supplementary References
