# Supplementary material for: Snf1/AMP-activated protein kinase activates Arf3p to promote invasive yeast growth via a non-canonical GEF domain
Source: Nat Commun. 2015 Jul 22;6:7840. doi: 10.1038/ncomms8840 (PMC4525183; doi:10.1038/ncomms8840)
Supplement: Supplementary Information — Supplementary Figures 1-14, Supplementary Tables 1-2 and Supplementary References [file ncomms8840-s1.pdf]

Supplementary Figure 1

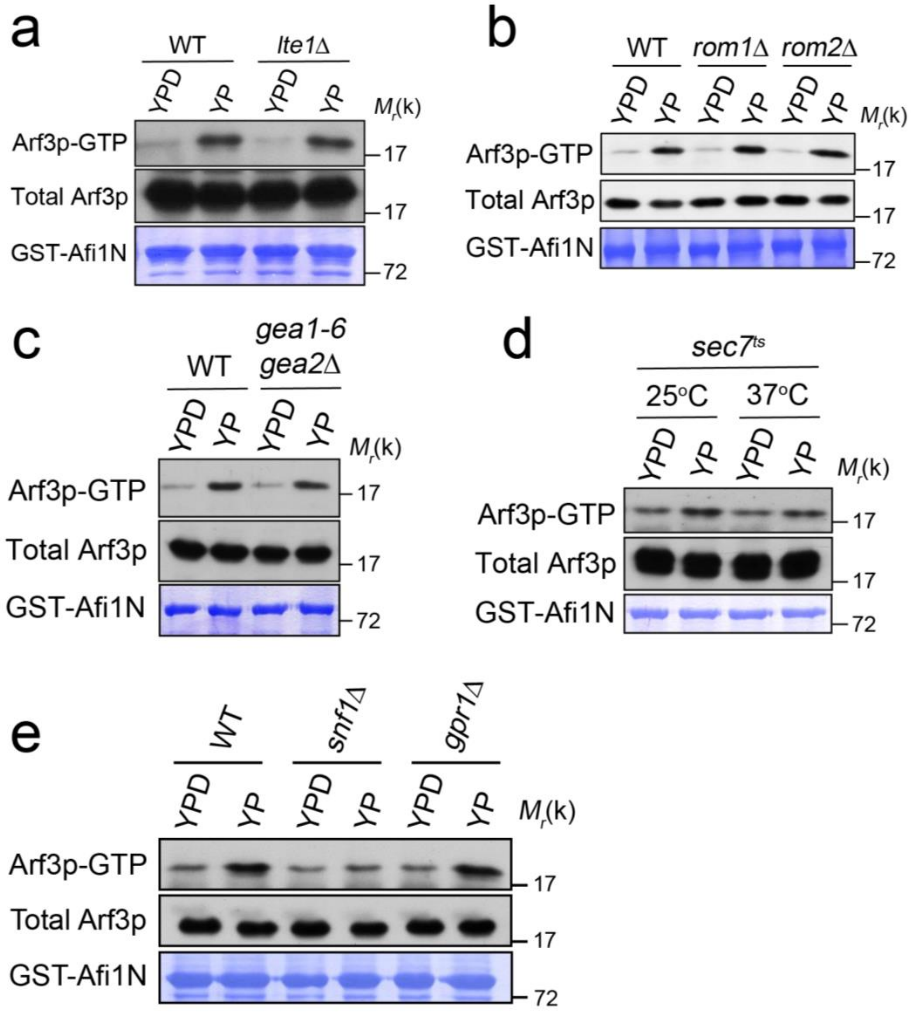

Supplementary Figure 1 | Activated Arf3p-GTP in indicated yeast cells in response to glucose depletion. (a-e) Indicated yeast cells grown in rich medium containing 2% glucose (YPD) were transferred to rich medium without glucose (YP) or rich medium with 2% glucose (YPD) for 2 h. Activated Arf3p-GTP was pulled-down from indicated cells using GST-Afi1N in and immunoblotted for Arf3p.

Supplementary Figure 2

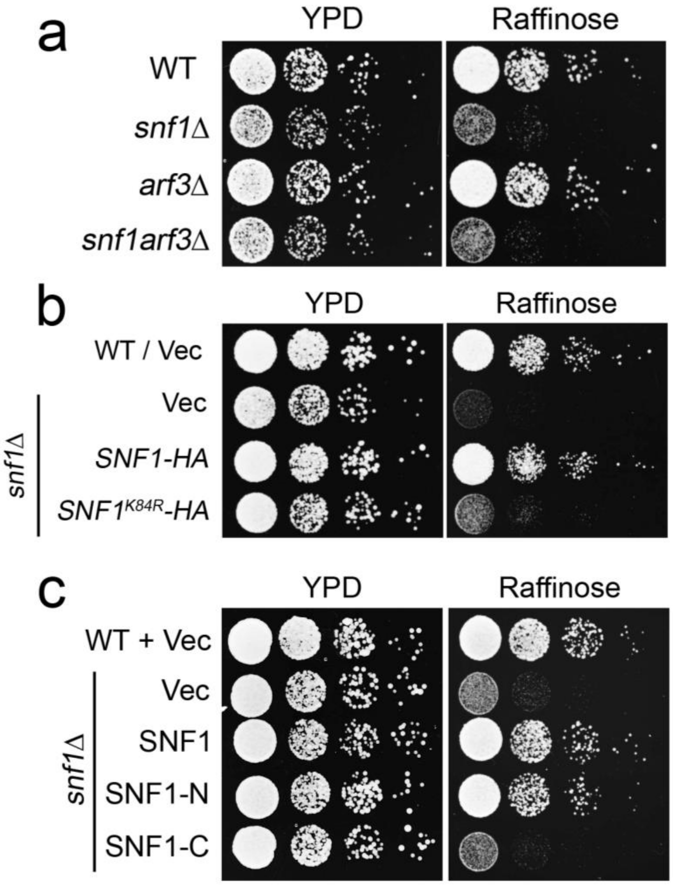

Supplementary Figure 2 | Raffinose hypersensitivity. (a-c) Serial dilutions of the strains indicated were spotted on YP medium containing 2% glucose (YPD) or 2% raffinose as the carbon source and grown for 3 days.

Supplementary Figure 3

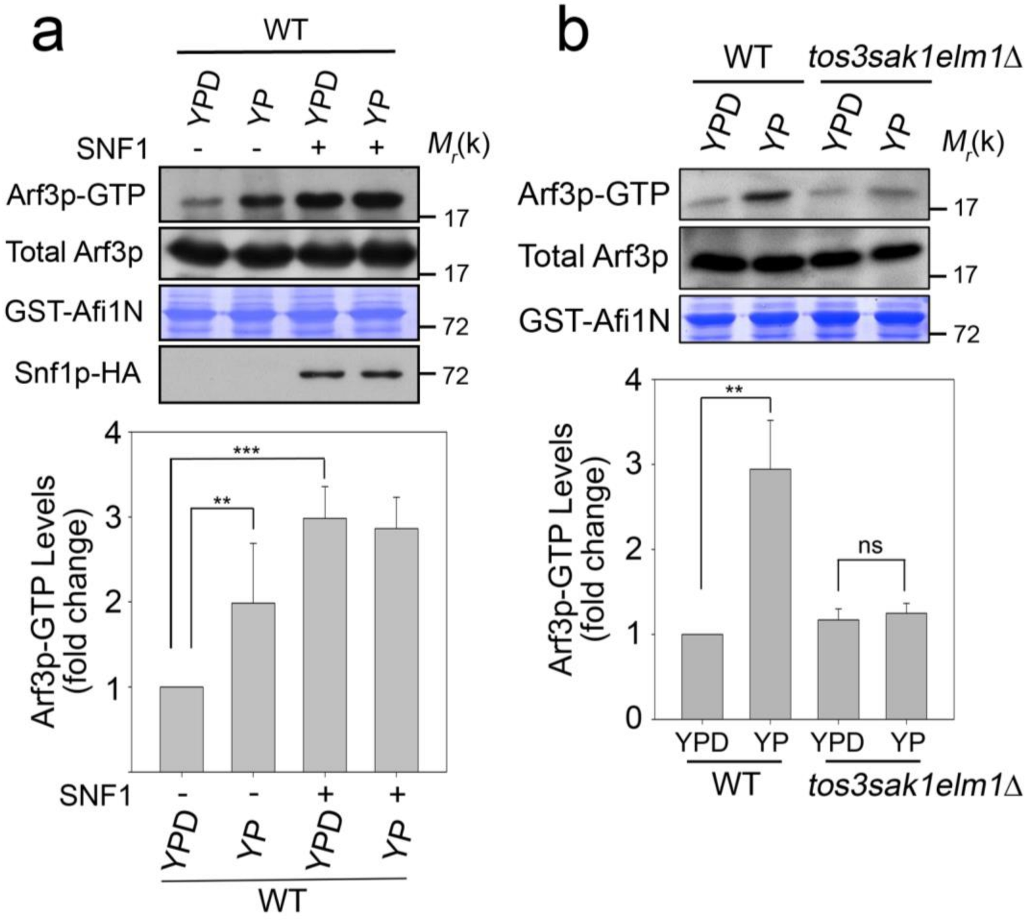

Supplementary Figure 3 | Activation of Snf1p is required for Arf3p activation. (a-b) Active forms of Arf3p were precipitated by GST-Afi1N in wild-type cells over-expressing *SNF1* **(a)** and in wild-type and *tos3sak1elm1*Δ cells **(b)** with YPD or YP treatment for 2 h. Snf1p has been suggested to be activated by upstream kinases or autophosphorylation when it is over-expressed in cells[1,2]. Below, quantitative analysis of active Arf3p. Data are reported as the mean ± S.D. of three experiments relative to **(a)** vector control and **(b)** wild-type in YPD. **, p<0.01; ***, p<0.001, Student's t-test.

Supplementary Figure 4

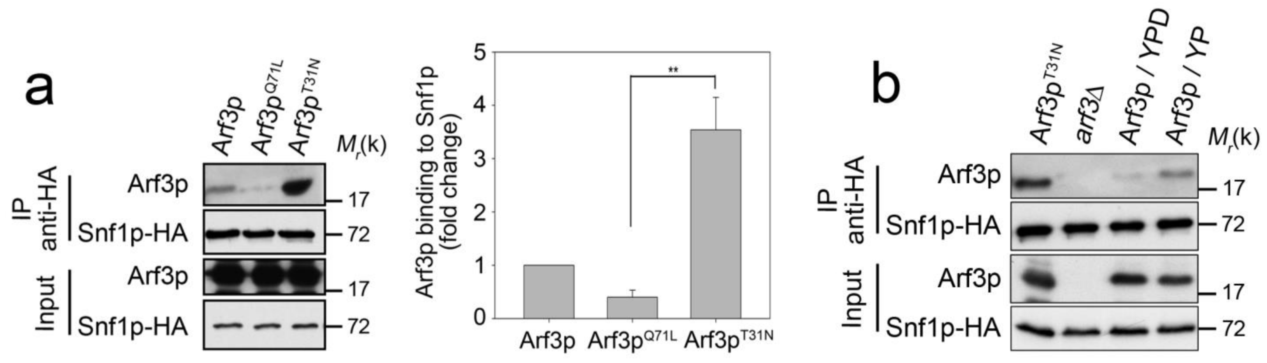

Supplementary Figure 4 | Snf1p interacts with inactive Arf3p *in vivo*. (a) Snf1p-HA was immunoprecipitated with anti-HA antibodies, and bound proteins were assayed for the presence of different forms of Arf3p. Data are reported as the mean ± S.D. of three experiments relative to wild-type Arf3p. **, $p<0.01$, Student's *t*-test. **(b)** Yeast cells were subjected to glucose depletion for 2 h; whole lysates were immunoprecipitated with anti-HA antibodies and immunoblotted for Arf3p.

Supplementary Figure 5

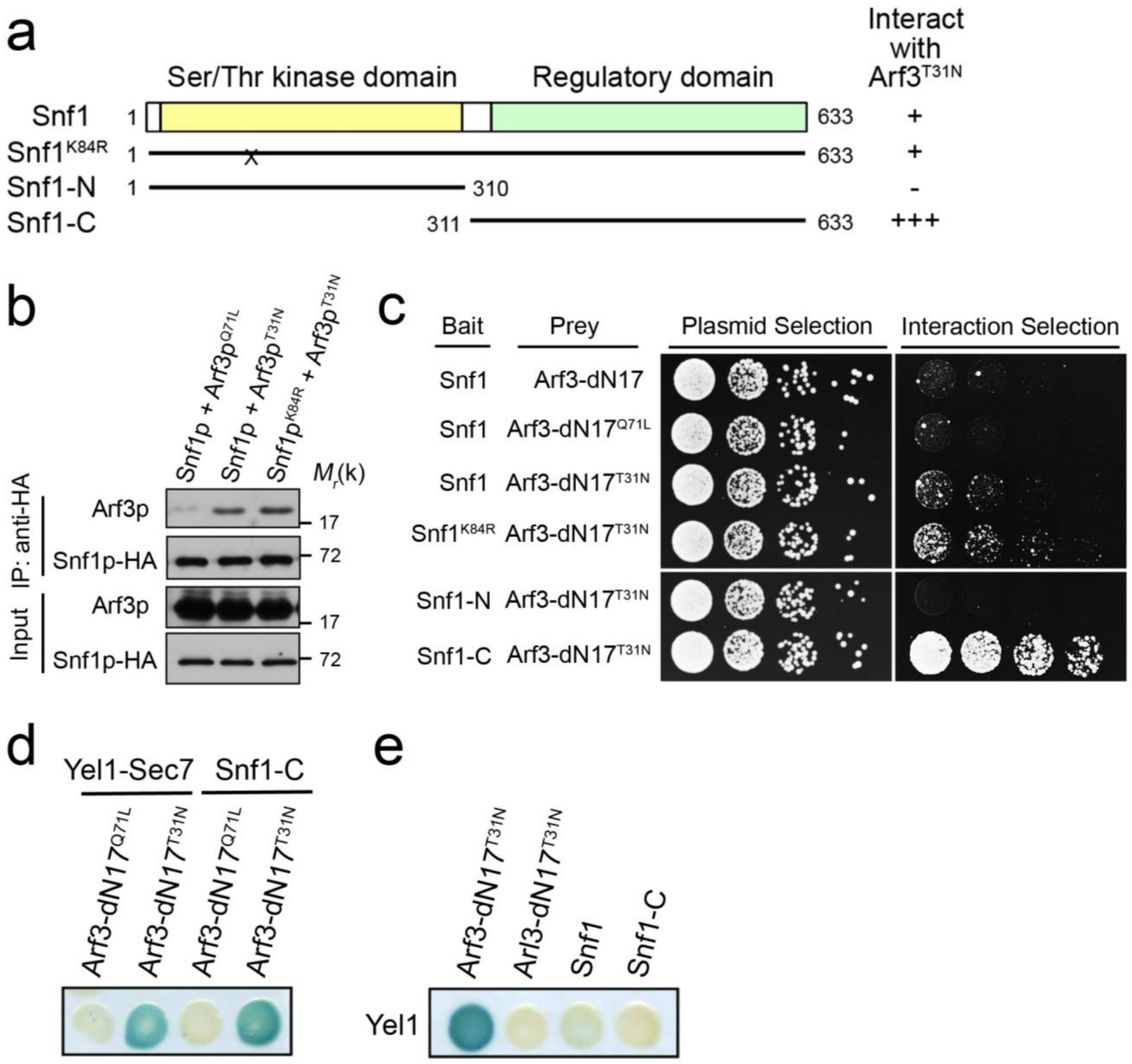

Supplementary Figure 5 | Snf1-C interacts with inactive forms of Arf3. (a) Snf1p fragments used in the experiments. **(b)** Lysates from yeast cells expressing Snf1p-HA immunoprecipitated with anti-HA antibodies and immunoblotted with anti-Arf3p antibodies. **(c-e)** Protein-protein interaction analysis of Snf1 by yeast two-hybrid analysis. Bait plasmids (pEG202) containing various forms of *ARF3-dN17* (wild-type, Q71L, and T31N) were co-transformed with pJG4-5 containing *SNF1*, *SNF1*^{K84R}, *SNF1-N*, *SNF1-C* **(c)** *YEL1* **(d)** and *YEL1-Sec7* **(e)** into yeast YEM1α and their interactions were analyzed using nutrient selection or β-galactosidase assay.

Supplementary Figure 6

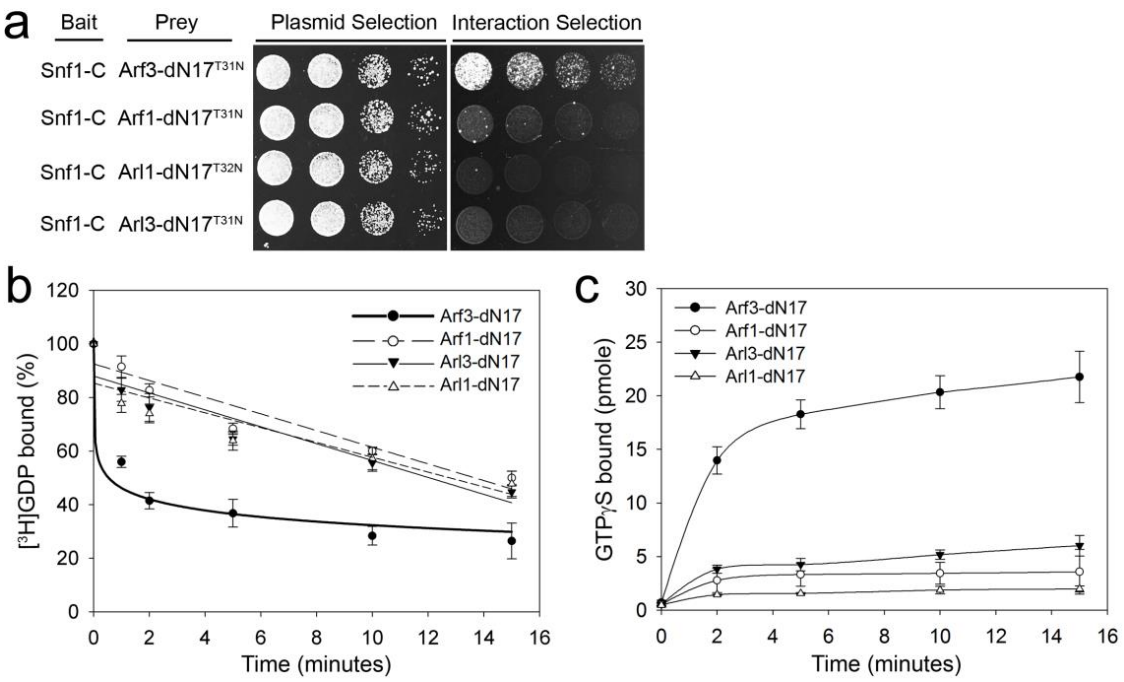

Supplementary Figure 6 | C-terminal regulatory domain of Snf1 specifically activates Arf3 *in vitro*. (a) Interactions between Snf1-C and Arf proteins were detected by yeast two-hybrid analysis. **(b-c)** $[^3H]$GDP dissociation **(b)** from and $[^{35}S]$GTPγS binding **(c)** to indicated Arf proteins in the presence of Snf1-C were monitored by measuring radioactivity. Data are reported as the mean ± S.D. of the percentage of $[^3H]$GDP dissociation **(b)** and of bound $[^{35}S]$GTPγS **(c)** (n=3).

Supplementary Figure 7

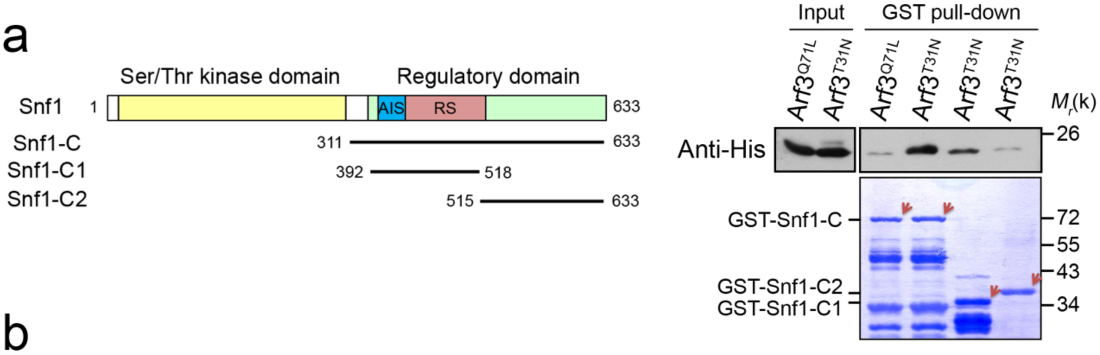

b

```
Rat_AMPKa2        -----------YDANVIDDEAVKEVCE--KFECTESEVMNSLYSGD---PQDQLAVAYHL 326
Mouse_AMPKa2      -----------YDANVIDDEAVKEVCE--KFECTESEVMNSLYSGD---PQDQLAVAYHL 326
Human_AMPKa2      -----------YDANVIDDEAVKEVCE--KFECTESEVMNSLYSGD---PQDQLAVAYHL 326
Worm_AMPKa2       -----------SEASIVDIEAVREVTEFQRYHVAEEEVTSALLGDD---PHHHLSIAYNL 399
Fly_SNF1A         -----------QDSNVIDTYAVAEVCT--KFGVKETEVHNSLLSGD---PHDQLAIAYHL 338
Yeast_SNF1        ENNDSKKDGSSPDNDEIDDNLVNILSS--TMGYEKDEIYESLESSEDTPAFNEIRDAYML 385
                              : . :*   *  :         : *: .:* ..:   . ..:  ** *

Rat_AMPKa2        IIDNRRIMNQAS---------EFYLASSPPT-------------------------GSFM 352
Mouse_AMPKa2      IIDNRRIMNQAS---------EFYLASSPPS-------------------------GSFM 352
Human_AMPKa2      IIDNRRIMNQAS---------EFYLASSPPS-------------------------GSFM 352
Worm_AMPKa2       IVDNKRIADETA---------KLSIEE-------------------------------FY 419
Fly_SNF1A         IIDNKRFADDAAN--QINEINNFFVAGSPPPPPPPPVPQSSMDHQAPLATVTVGGGTSAS 396
Yeast_SNF1        IKENKSLIKDMKANKSVSDELDTFLSQSPPT---------------------------FQ 418
                  * :*: : .:           .  :

Rat_AMPKa2        DDMAMHIPP----------GLKPHPERMPPL-------------------------IADS 377
Mouse_AMPKa2      DDSAMHIPP----------GLKPHPERMPPL-------------------------IADS 377
Human_AMPKa2      DDSAMHIPP----------GLKPHPERMPPL-------------------------IADS 377
Worm_AMPKa2       QVTPNKGPG----------PVHRHPER-----------------------------IAAS 440
Fly_SNF1A         SGTATPVPPVAGGTPSSTIPIRPHPERIAPMRDRQ---------------------LAMS 435
Yeast_SNF1        QQSKSHQKS-----QVDHETAKQHARRMASAITQQRTYHQSPFMDQYKEEDSTVSILPTS 473
                  .                    : *..*                             :. *

Rat_AMPKa2        PKARCPLDALNTTKPKSLAVK-------KAKWHLGIRSQSKPYDIMAEVYRAMKQLDFEW 430
Mouse_AMPKa2      PKARCPLDALNTTKPKSLAVK-------KAKWHLGIRSQSKACDIMAEVYRAMKQLGFEW 430
Human_AMPKa2      PKARCPLDALNTTKPKSLAVK-------KAKWHLGIRSQSKPYDIMAEVYRAMKQLDFEW 430
Worm_AMPKa2       VSSKITPTLDNTEASGANRNK-------RAKWHLGIRSQSRPEDIMFEVFRAMKQLDMEW 493
Fly_SNF1A         VQTSGGGAFPEKTARGGTPIK-------RAKWHLGIRSQSKPNDIMLEVYRAMKALSYEW 488
Yeast_SNF1        LPQIHRANMLAQGSPAASKISPLVTKKSKTRWHFGIRSRSYPLDVMGEIYIALKNLGAEW 533
                                  .   .       :::**:****:* . *:* *:: *:* *. **
```

c

Name	Sequence	Interaction with Arf3^{T31N}	Raffinose Sensitivity	GEF Activity
SNF1-AIS	^{359} EKDEIYESLESSEDTPAFNEIRDAYMLIKE ^{388}	+	+	+
SNF1-AISm1	^{359} AAAAAYESLESSEDTPAFNEIRDAYMLIKE ^{388}	+	+	+
SNF1-AISm2	^{359} EKDEIAAAAASSEDTPAFNEIRDAYMLIKE ^{388}	+	+	+
SNF1-AISm3	^{359} EKDEIYESLEAAAAAPAFNEIRDAYMLIKE ^{388}	+	+	+
SNF1-AISm4	^{359} EKDEIYESLESSEDTAAAAAIRDAYMLIKE ^{388}	+	+	+
SNF1-AISm5	^{359} EKDEIYESLESSEDTPAFNEAAAAAMLIKE ^{388}	+	+	+
SNF1-AISm6	^{359} EKDEIYESLESSEDTPAFNEIRDAYAAAAA ^{388}	+	+	+
SNF1-RS	^{500} KSKTRWHFGIRSRSYPLDVMGEIYIALKNLGAEWA ^{530}	+	+	+
SNF1-A1	^{500} AAAAAWHFGIRSRSYPLDVMGEIYIALKNLGAEWA ^{530}	+	+	+
SNF1-A2	^{500} KSKTRAAAAARSRSYPLDVMGEIYIALKNLGAEWA ^{530}	+	-	-
SNF1-A3	^{500} KSKTRWHFGIAAAAAPLDVMGEIYIALKNLGAEWA ^{530}	+	+	+
SNF1-A4	^{500} KSKTRWHFGIRSRSYAAAAAGEIYIALKNLGAEWA ^{530}	+	+	+
SNF1-A5	^{500} KSKTRWHFGIRSRSYPLDVMAAAAAALKNLGAEWA ^{530}	-	+	-
SNF1-A6	^{500} KSKTRWHFGIRSRSYPLDVMGEIYIAAAAAGAEWA ^{530}	+	+	+
SNF1-A7	^{500} KSKTRWHFGIRSRSYPLDVMGEIYIALKNLAAAAA ^{530}	+	+	+

Supplementary Figure 7 | Supplementary Figure 4. Interaction between Snf1 and Arf3. (a) Interactions between Snf1-C fragment sequences with Arf3 were tested by *in vitro* binding assay as described in Methods **(b)** Alignment of C1 fragment of Snf1p with other members of AMPK protein family. The sequences were aligned using CLUSTALW and the markings indicate that more than half of the residues are highly related (:), semi-conserved (.), or identical (*). (**c)** Summary of effects of indicated alanine mutations at the Snf1p regulatory domain.

Supplementary Figure 8

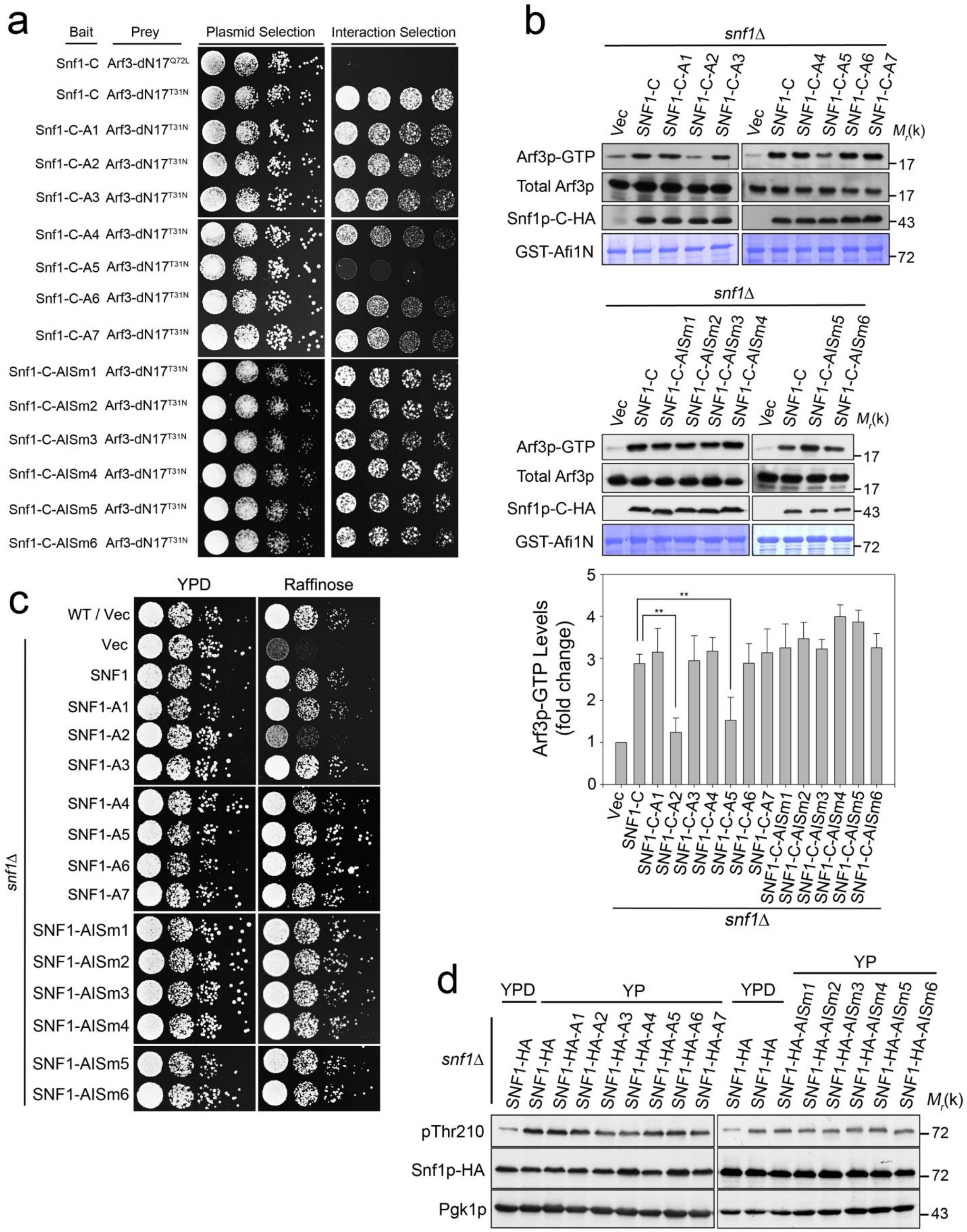

Supplementary Figure 8 | Functional analysis of Snf1p mutant proteins. (a) Serial mutagenesis strategy for mapping the Snf1 and Arf3 interaction surface by yeast two-hybrid assay. **(b)** Arf3p-GTP forms were

precipitated by GST-Afi1N in *snf1*Δ cells expressing C-terminal fragments of Snf1p variants. Data are reported as the mean fold change ± S.D. of three experiments relative to vector control. **, $p<0.01$ **(c)** Serial dilutions of the strains indicated were spotted on YP medium containing 2% glucose (YPD) or 2% raffinose as the carbon source and grown for 3 days. **(d)** Phosphorylation of residue Thr210 of Snf1p mutant proteins was detected by specific antibodies (Cell Signaling).

Supplementary Figure 9

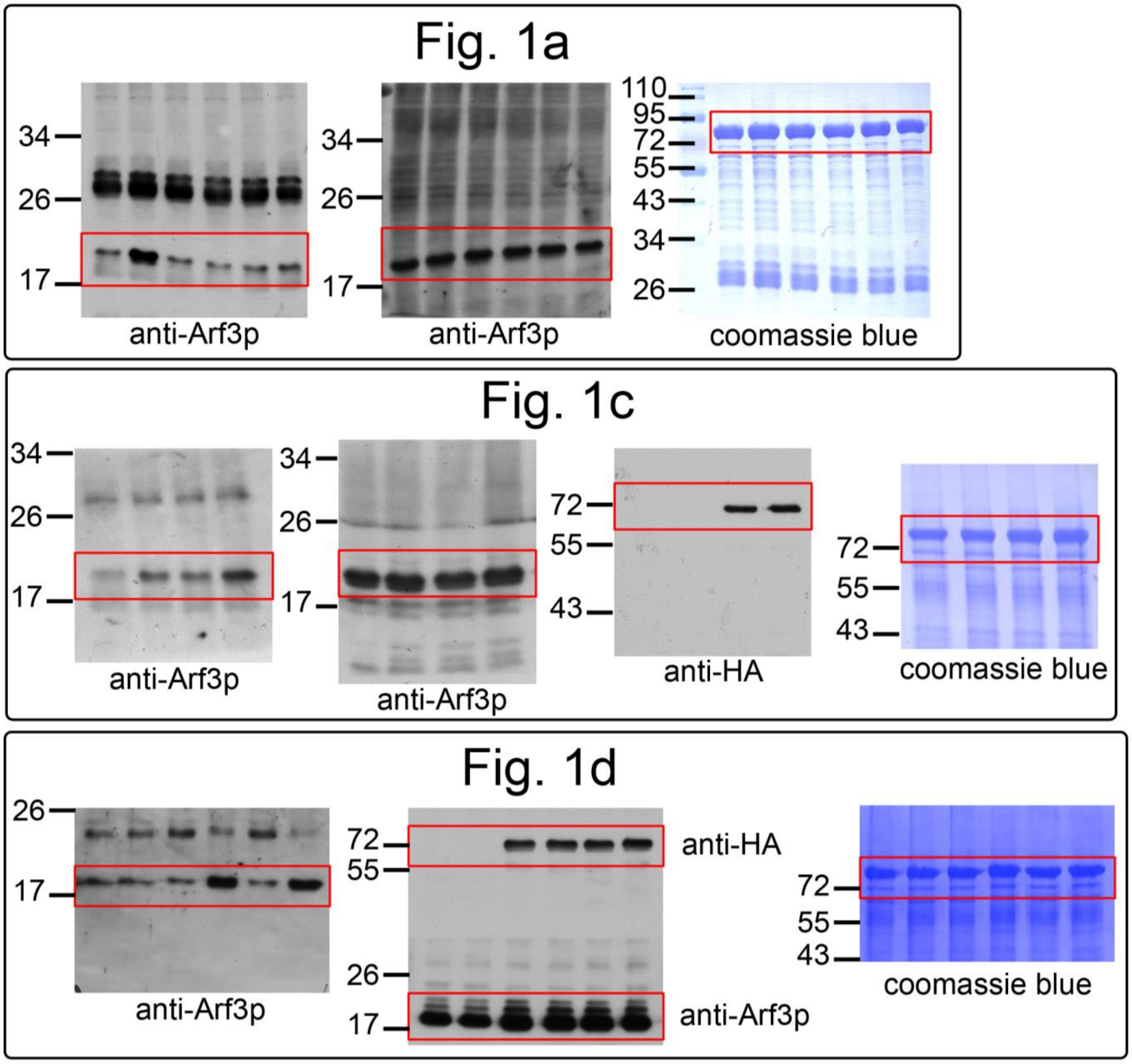

Supplementary Figure 9 | Uncropped images of the original scans of immunoblots. Uncropped, full-size scans of immunoblots shown in Fig. 1a, 1c-1d.

Supplementary Figure 10

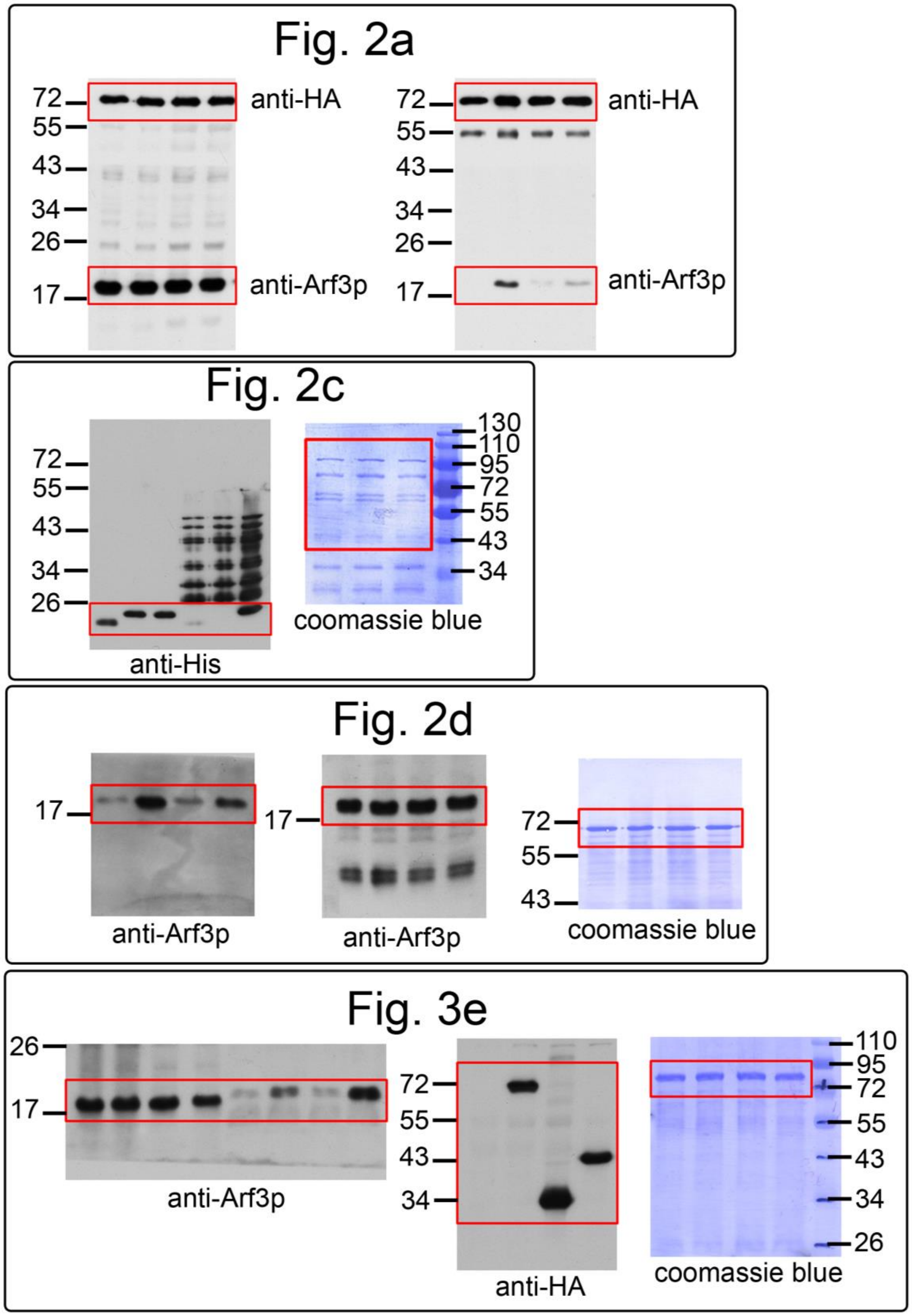

Supplementary Figure 10 | Uncropped images of the original scans of immunoblots. Uncropped, full-size scans of immunoblots shown in Fig. 2a, 2c-2d and 3e.

Supplementary Figure 11

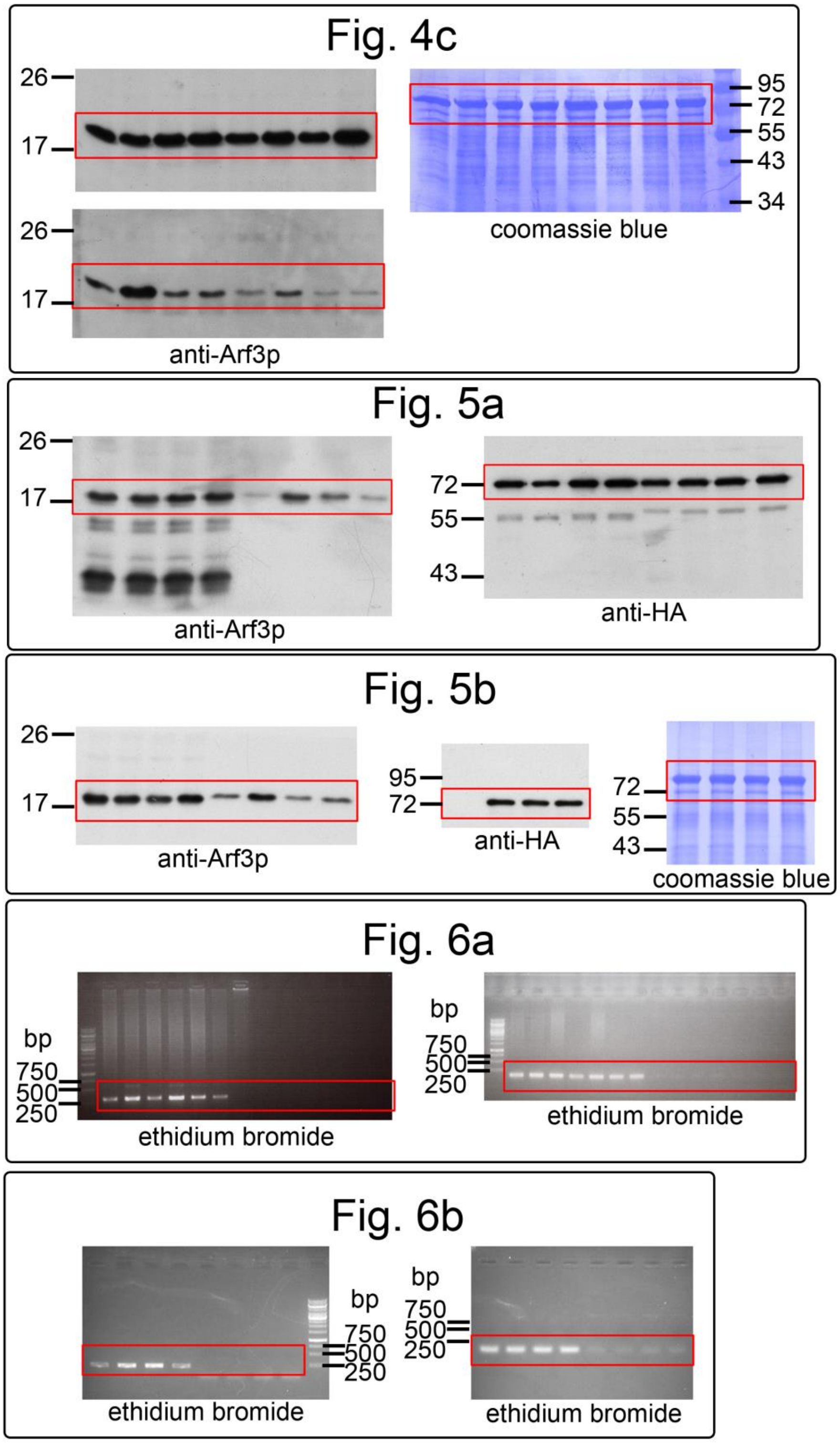

Supplementary Figure 11 | Uncropped images of the original scans of immunoblots. Uncropped, full-size scans of immunoblots shown in Fig. 4c, 5a-5b and 6a-6b.

Supplementary Figure 12

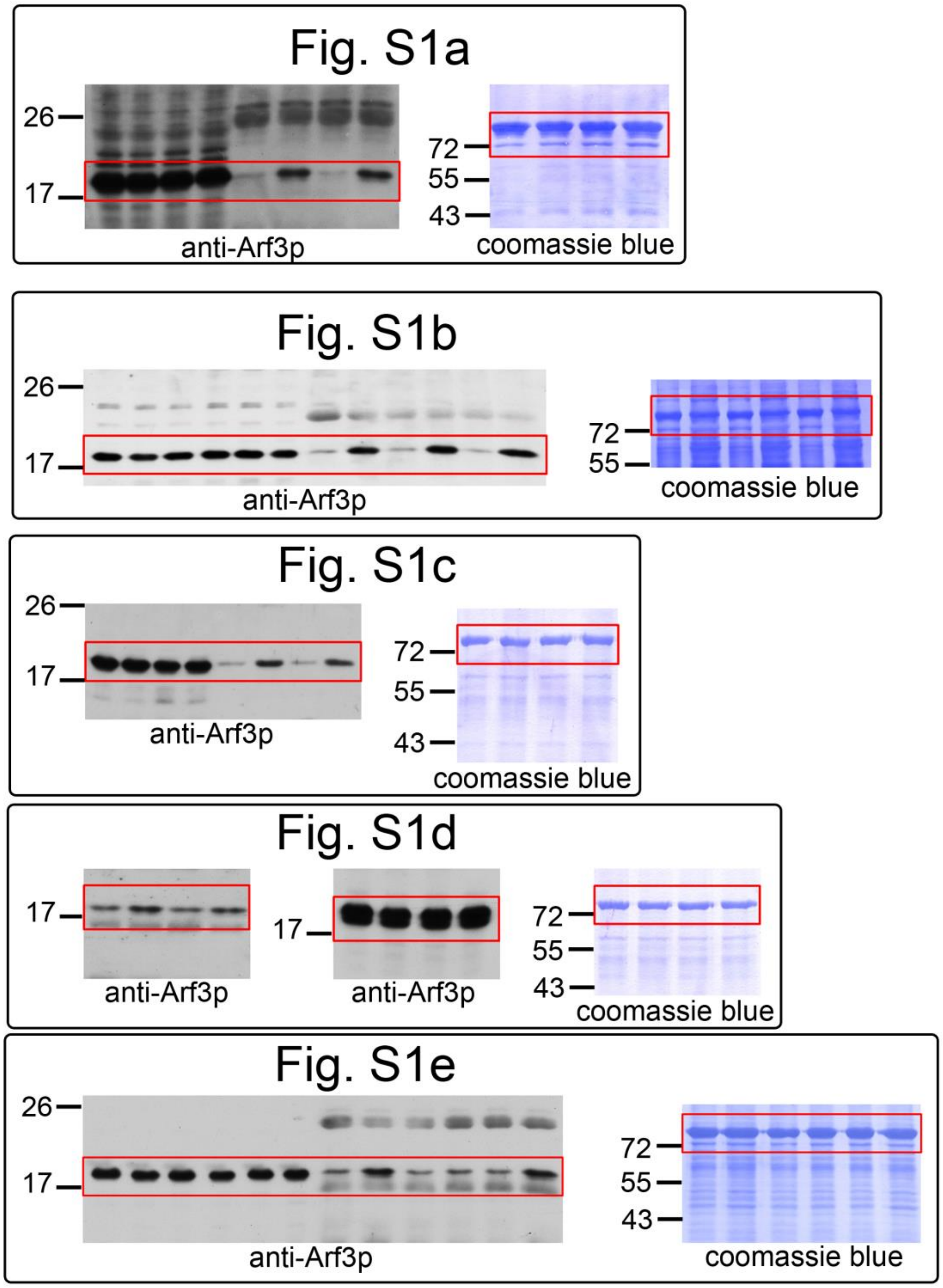

Supplementary Figure 12 | Uncropped images of the original scans of immunoblots. Uncropped, full-size scans of immunoblots shown in Supplementary Fig. 1a-1e.

Supplementary Figure 13

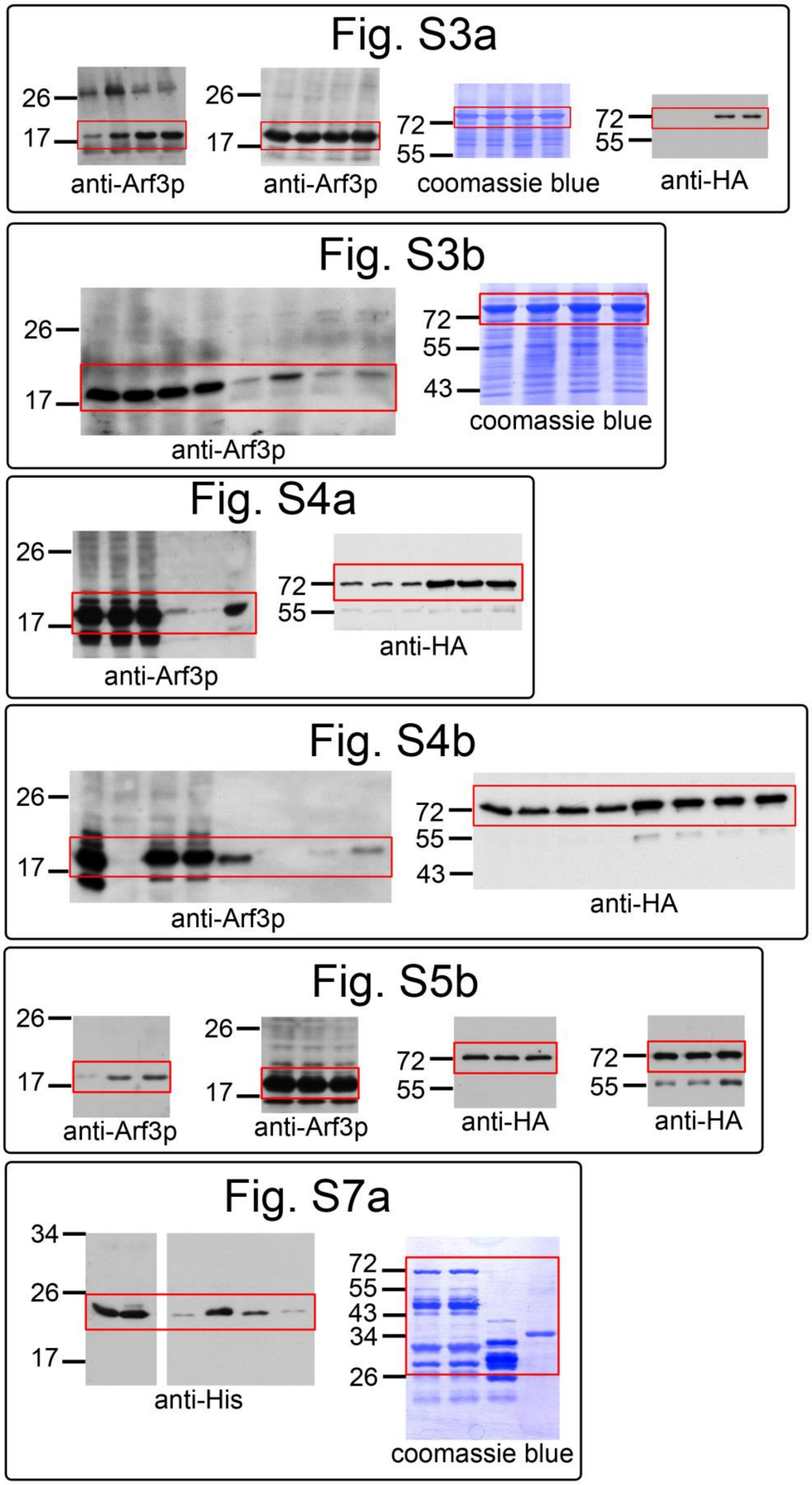

Supplementary Figure 13 | Uncropped images of the original scans of immunoblots. Uncropped, full-size scans of immunoblots shown in Supplementary Fig. 3a-3b, 4a-4b, 5b and 7a.

Supplementary Figure 14

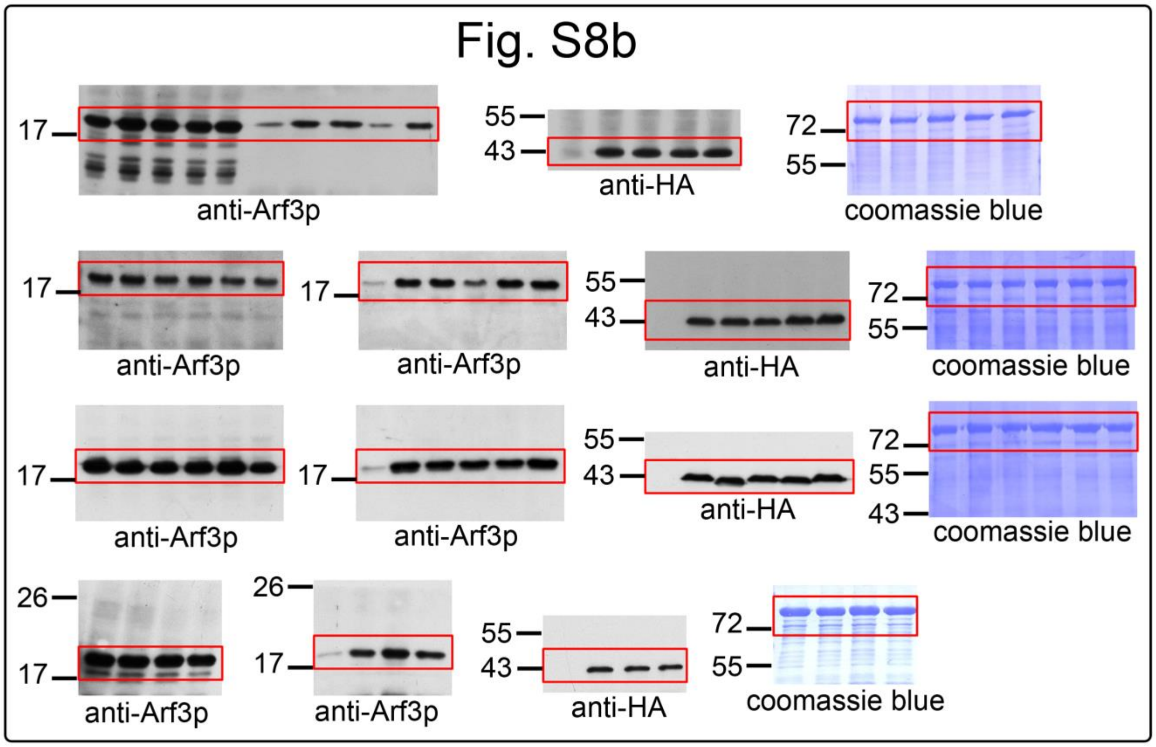

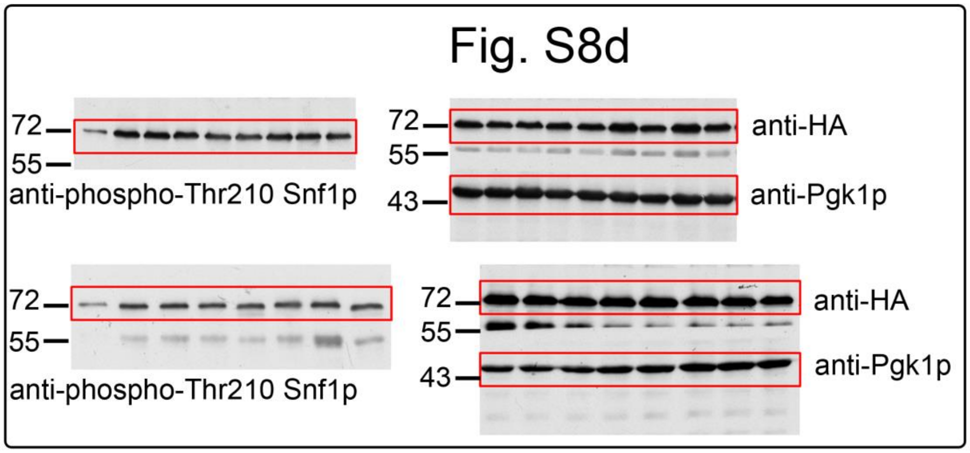

Supplementary Figure 14 | Uncropped images of the original scans of immunoblots. Uncropped, full-size scans of immunoblots shown in Supplementary Fig. 8b and 8d.

Supplementary Tables

Supplementary Table 1 | Yeast strains used in this study

Strain	Genotype	Reference
Σ1278b 10560-4A	*MATa, ura3-52, leu2::hisG, trp1::hisG, his3::hisG*	[3]
Σ1278b 10560-4A *arf3*Δ	*MATa, ura3-52, leu2::hisG, trp1::hisG, his3::hisG, arf3::KanMX6*	[4]
Σ1278b 10560-4A *yel1*Δ	*MATa, ura3-52, leu2::hisG, trp1::hisG, his3::hisG, yel1::KanMX6*	[4]
Σ1278b 10560-4A *snf1*Δ	*MATa, ura3-52, leu2::hisG, trp1::hisG, his3::hisG, snf1::His3MX6*	This study
Σ1278b 10560-4A *yel1snf1*Δ	*MATa, ura3-52, leu2::hisG, trp1::hisG, his3::hisG, yel1::KanMX6, snf1::His3MX6*	This study
Σ1278b 10560-4A *snf1arf3*Δ	*MATa, ura3-52, leu2::hisG, trp1::hisG, his3::hisG, snf1::His3MX6, arf3::KanMX6*	This study
Σ1278b 10560-4A *flo11*Δ	*MATa, ura3-52, leu2::hisG, trp1::hisG, his3::hisG, flo11::KanMX6*	[4]
Σ1278b 10560-4A *gpr1*Δ	*MATa, ura3-52, leu2::hisG, trp1::hisG, his3::hisG, gpr1::His3MX6*	This study
Σ1278b 10560-4A *lte1*Δ	*MATa, ura3-52, leu2::hisG, trp1::hisG, his3::hisG, lte1::His3MX6*	This study
Σ1278b 10560-4A *rom1*Δ	*MATa, ura3-52, leu2::hisG, trp1::hisG, his3::hisG, rom1::His3MX6*	This study
Σ1278b 10560-4A *rom2*Δ	*MATa, ura3-52, leu2::hisG, trp1::hisG, his3::hisG, rom2::His3MX6*	This study
Σ1278b 10560-4A *syt1*Δ	*MATa, ura3-52, leu2::hisG, trp1::hisG, his3::hisG, syt1::His3MX6*	This study
CLJ815	*MATα, ura3-52, his3-Δ200, leu2-3, -122trp1-Δ901, suc2-Δ9, lys2-801, gea1-6, gea2::HIS3*	[5]
sec7[ts]	*MATα, leu2, ura3-1, his3,11-15, sec7-4*	
YEM1α	*MATα, trp1, his3, leu2, 6ops-LEU2, 2ops-LacZ*	
W303-1A	*MATa, ura3, his3, leu2, trp1, ade2, can1*	[6]
*tos3sak1elm1*Δ	*MATa, ura3, his3, leu2, trp1, ade2, can1, tos3::kanMX4, sak1::kanMX4, elm1::URA3*	[6]

Supplementary Table 2 | Yeast plasmids used in this study

Plasmid	Description	Reference
pJG4-5	*2-µm, TRP1, GAL1p*	[7]
pJG4-5-SNF1	*2-µm, TRP1, GAL1p-SNF1*	This study
pJG4-5-SNF1^{K84R}	*2-µm, TRP1, GAL1p-SNF1*^{K84R}	This study
pJG4-5-SNF1-N	*2-µm, TRP1, GAL1p-SNF1-N1-310*	This study
pJG4-5-SNF1-C	*2-µm, TRP1, GAL1p-SNF1-C311-633*	This study
pJG4-5-SNF1-C-A1	*2-µm, TRP1, GAL1p-SNF1-C-500-504A*	This study
pJG4-5-SNF1-C-A2	*2-µm, TRP1, GAL1p-SNF1-C-505-509A*	This study
pJG4-5-SNF1-C-A3	*2-µm, TRP1, GAL1p-SNF1-C-510-514A*	This study
pJG4-5-SNF1-C-A4	*2-µm, TRP1, GAL1p-SNF1-C-515-519A*	This study
pJG4-5-SNF1-C-A5	*2-µm, TRP1, GAL1p-SNF1-C-520-524A*	This study
pJG4-5-SNF1-C-A6	*2-µm, TRP1, GAL1p-SNF1-C-525-529A*	This study
pJG4-5-SNF1-C-A7	*2-µm, TRP1, GAL1p-SNF1-C-530-534A*	This study
pJG4-5-SNF1-C-AISm1	*2-µm, TRP1, GAL1p-SNF1-C-359-363A*	This study
pJG4-5-SNF1-C-AISm2	*2-µm, TRP1, GAL1p-SNF1-C-364-368A*	This study
pJG4-5-SNF1-C-AISm3	*2-µm, TRP1, GAL1p-SNF1-C-369-373A*	This study
pJG4-5-SNF1-C-AISm4	*2-µm, TRP1, GAL1p-SNF1-C-374-378A*	This study
pJG4-5-SNF1-C-AISm5	*2-µm, TRP1, GAL1p-SNF1-C-379-383A*	This study
pJG4-5-SNF1-C-AISm6	*2-µm, TRP1, GAL1p-SNF1-C-384-388A*	This study
pJG4-5-YEL1	*2-µm, TRP1, GAL1p-YEL1*	[8]
pJG4-5-YEL1-Sec7	*2-µm, TRP1, GAL1p-YEL1-Sec7*	This study
pEG202	*2-µm, HIS3, ADH1p*	[7]
pEG202-ARF3	*2-µm, HIS3, ADH1p-ARF3-dN17*	[9]
pEG202-ARF3^{Q71L}	*2-µm, HIS3, ADH1p-ARF3*^{Q71L}*-dN17*	[9]
pEG202-ARF3^{T31N}	*2-µm, HIS3, ADH1p-ARF3*^{T31N}*-dN17*	[9]
pEG202-ARF1^{T31N}	*2-µm, HIS3, ADH1p-ARF1*^{T31N}*-dN17*	[10]
pEG202-ARL1^{T32N}	*2-µm, HIS3, ADH1p-ARL1*^{T32N}*-dN17*	[10]
pEG202-ARL3^{T31N}	*2-µm, HIS3, ADH1p-ARL3*^{T31N}*-dN17*	[10]
pVT101U	*2-µm, URA3, ADH1p*	[11]
pVT101U-SNF1-HA	*2-µm, URA3, ADH1p-SNF1-HA*	This study
pVT101U-SNF1^{K84R}-HA	*2-µm, URA3, ADH1p-SNF1*^{K84R}*-HA*	This study
pVT101U-YEL1	*2-µm, URA3, ADH1p-YEL1*	This study
pVT101U-SNF1-N-HA	*2-µm, URA3, ADH1p-SNF1-N1-310-HA*	This study
pVT101U-SNF1-C-HA	*2-µm, URA3, ADH1p-SNF1-C311-633-HA*	This study
pVT101U-SNF1-A1-HA	*2-µm, URA3, ADH1p-SNF1-HA-500-504A*	This study
pVT101U-SNF1-A2-HA	*2-µm, URA3, ADH1p-SNF1-HA-505-509A*	This study

pVT101U-SNF1-A3-HA	*2-μm, URA3, ADH1p-SNF1-HA-510-514A*	This study
pVT101U-SNF1-A4-HA	*2-μm, URA3, ADH1p-SNF1-HA-515-519A*	This study
pVT101U-SNF1-A5-HA	*2-μm, URA3, ADH1p-SNF1-HA-520-524A*	This study
pVT101U-SNF1-A6-HA	*2-μm, URA3, ADH1p-SNF1-HA-525-529A*	This study
pVT101U-SNF1-A7-HA	*2-μm, URA3, ADH1p-SNF1-HA-530-534A*	This study
pVT101U-SNF1-AISm1-HA	*2-μm, URA3, ADH1p-SNF1-HA-359-363A*	This study
pVT101U-SNF1-AISm2-HA	*2-μm, URA3, ADH1p-SNF1-HA-364-368A*	This study
pVT101U-SNF1-AISm3-HA	*2-μm, URA3, ADH1p-SNF1-HA-369-373A*	This study
pVT101U-SNF1-AISm4-HA	*2-μm, URA3, ADH1p-SNF1-HA-374-378A*	This study
pVT101U-SNF1-AISm5-HA	*2-μm, URA3, ADH1p-SNF1-HA-379-383A*	This study
pVT101U-SNF1-AISm6-HA	*2-μm, URA3, ADH1p-SNF1-HA-384-388A*	This study
pVT101U-SNF1-C-A1	*2-μm, URA3, ADH1p-SNF1- C-500-504A-HA*	This study
pVT101U-SNF1-C-A2	*2-μm, URA3, ADH1p-SNF1- C-505-509A-HA*	This study
pVT101U-SNF1-C-A3	*2-μm, URA3, ADH1p-SNF1- C-510-514A-HA*	This study
pVT101U-SNF1-C-A4	*2-μm, URA3, ADH1p-SNF1- C-515-519A-HA*	This study
pVT101U-SNF1-C-A5	*2-μm, URA3, ADH1p-SNF1- C-520-524A-HA*	This study
pVT101U-SNF1-C-A6	*2-μm, URA3, ADH1p-SNF1- C-525-529A-HA*	This study
pVT101U-SNF1-C-A7	*2-μm, URA3, ADH1p-SNF1- C-530-534A-HA*	This study
pVT101U-SNF1-C-AISm1	*2-μm, URA3, ADH1p-SNF1-C-359-363A*	This study
pVT101U-SNF1-C-AISm2	*2-μm, URA3, ADH1p-SNF1-C-364-368A*	This study
pVT101U-SNF1-C-AISm3	*2-μm, URA3, ADH1p-SNF1-C-369-373A*	This study
pVT101U-SNF1-C-AISm4	*2-μm, URA3, ADH1p-SNF1-C-374-378A*	This study
pVT101U-SNF1-C-AISm5	*2-μm, URA3, ADH1p-SNF1-C-379-383A*	This study
pVT101U-SNF1-C-AISm6	*2-μm, URA3, ADH1p-SNF1-C-384-388A*	This study
YEplac181	*2-μm, LEU2*	[12]
YEplac181-ARF3	*2-μm, LEU2, ARF3p-ARF3*	[4]
YEplac181-ARF3^{Q71L}	*2-μm, LEU2, ARF3p-ARF3*^{Q71L}	[4]
YEplac181-ARF3^{T31N}	*2-μm, LEU2, ARF3p-ARF3*^{T31N}	[4]
YCplac111	*CEN, LEU2*	[12]
YCplac111-ARF3-GFP	*CEN, LEU2, ADH1p-ARF3-GFP*	This study
YIplac211	*URA3*	[12]
YIplac211-SNF1-HA	*URA3, ADH1p-SNF1-HA*	This study
YIplac211- SNF1^{K84R}-HA	*URA3, ADH1p-SNF1*^{K84R}*-HA*	This study

References

1. Baena-Gonzalez, E., Rolland, F., Thevelein, J. M. & Sheen, J. A central integrator of transcription networks in plant stress and energy signalling. *Nature* **448**, 938-942 (2007).
2. Kahn, B. B., Alquier, T., Carling, D. & Hardie, D. G. AMP-activated protein kinase: ancient energy gauge provides clues to modern understanding of metabolism. *Cell Metab* **1**, 15-25 (2005).
3. Lo, W. S. & Dranginis, A. M. The cell surface flocculin Flo11 is required for pseudohyphae formation and invasion by Saccharomyces cerevisiae. *Mol Biol Cell* **9**, 161-171 (1998).
4. Hsu, J. W. & Lee, F. J. Arf3p GTPase is a key regulator of Bud2p activation for invasive growth in Saccharomyces cerevisiae. *Mol Biol Cell* **24**, 2328-2339 (2013).
5. Spang, A., Herrmann, J. M., Hamamoto, S. & Schekman, R. The ADP ribosylation factor-nucleotide exchange factors Gea1p and Gea2p have overlapping, but not redundant functions in retrograde transport from the Golgi to the endoplasmic reticulum. *Mol Biol Cell* **12**, 1035-1045 (2001).
6. Hong, S. P., Leiper, F. C., Woods, A., Carling, D. & Carlson, M. Activation of yeast Snf1 and mammalian AMP-activated protein kinase by upstream kinases. *Proc Natl Acad Sci U S A* **100**, 8839-8843 (2003).
7. Gyuris, J., Golemis, E., Chertkov, H. & Brent, R. Cdi1, a human G1 and S phase protein phosphatase that associates with Cdk2. *Cell* **75**, 791-803 (1993).
8. Tsai, P. C. *et al.* Afi1p functions as an Arf3p polarization-specific docking factor for development of polarity. *J Biol Chem* **283**, 16915-16927 (2008).
9. Huang, C. F., Liu, Y. W., Tung, L., Lin, C. H. & Lee, F. J. Role for Arf3p in development of polarity, but not endocytosis, in Saccharomyces cerevisiae. *Mol Biol Cell* **14**, 3834-3847 (2003).
10. Chen, K. Y. *et al.* Syt1p promotes activation of Arl1p at the late Golgi to recruit Imh1p. *J Cell Sci* **123**, 3478-3489 (2010).
11. Vernet, T., Dignard, D. & Thomas, D. Y. A family of yeast expression vectors containing the phage f1 intergenic region. *Gene* **52**, 225-233 (1987).
12. Gietz, R. D. & Sugino, A. New yeast-Escherichia coli shuttle vectors constructed with in vitro mutagenized yeast genes lacking six-base pair restriction sites. *Gene* **74**, 527-534 (1988).
